# Cardiomyocyte SORBS2 expression increases in heart failure and regulates integrin interactions and extracellular matrix composition

**DOI:** 10.1093/cvr/cvaf021

**Published:** 2025-02-17

**Authors:** Louk T Timmer, Elvira den Hertog, Danielle Versteeg, Harm Post, Job A J Verdonschot, Jantine Monshouwer-Kloots, Eirini Kyriakopoulou, Ilaria Perini, Tim Koopmans, Petra van der Kraak, Lorena Zentilin, Stephane R B Heymans, Aryan Vink, Mauro Giacca, Albert J R Heck, Eva van Rooij

**Affiliations:** Hubrecht Institute, Royal Netherlands Academy of Arts and Sciences (KNAW), University Medical Center Utrecht, Uppsalalaan 8, 3584 CT Utrecht, The Netherlands; Hubrecht Institute, Royal Netherlands Academy of Arts and Sciences (KNAW), University Medical Center Utrecht, Uppsalalaan 8, 3584 CT Utrecht, The Netherlands; Hubrecht Institute, Royal Netherlands Academy of Arts and Sciences (KNAW), University Medical Center Utrecht, Uppsalalaan 8, 3584 CT Utrecht, The Netherlands; Department of Cardiology, University Medical Center Utrecht, Heidelberglaan 100, 3584 CX Utrecht, The Netherlands; Biomolecular Mass Spectrometry and Proteomics Group, Bijvoet Center for Biomolecular Research, Utrecht Institute for Pharmaceutical Sciences, Utrecht University, Utrecht, The Netherlands; Netherlands Proteomics Center, Utrecht, The Netherlands; Department of Clinical Genetics, Maastricht University Medical Center, Maastricht, The Netherlands; Department of Cardiology, Cardiovascular Research Institute (CARIM), Maastricht University Medical Center, Maastricht, The Netherlands; European Reference Network for Rare, Low Prevalence and Complex Diseases of the Heart (ERN GUARD-Heart); Hubrecht Institute, Royal Netherlands Academy of Arts and Sciences (KNAW), University Medical Center Utrecht, Uppsalalaan 8, 3584 CT Utrecht, The Netherlands; Hubrecht Institute, Royal Netherlands Academy of Arts and Sciences (KNAW), University Medical Center Utrecht, Uppsalalaan 8, 3584 CT Utrecht, The Netherlands; Hubrecht Institute, Royal Netherlands Academy of Arts and Sciences (KNAW), University Medical Center Utrecht, Uppsalalaan 8, 3584 CT Utrecht, The Netherlands; Hubrecht Institute, Royal Netherlands Academy of Arts and Sciences (KNAW), University Medical Center Utrecht, Uppsalalaan 8, 3584 CT Utrecht, The Netherlands; Department of Pathology, University Medical Center Utrecht, Utrecht, The Netherlands; Molecular Medicine Laboratory, International Center for Genetic Engineering and Biotechnology (ICGEB), Trieste, Italy; Department of Cardiology, Cardiovascular Research Institute (CARIM), Maastricht University Medical Center, Maastricht, The Netherlands; Department of Cardiovascular Sciences, Center for Molecular and Vascular Biology, KU Leuven, Leuven, Belgium; Department of Pathology, University Medical Center Utrecht, Utrecht, The Netherlands; Molecular Medicine Laboratory, International Center for Genetic Engineering and Biotechnology (ICGEB), Trieste, Italy; School of Cardiovascular and Metabolic Medicine & Sciences, British Heart Foundation Centre of Research Excellence, King’s College London, London, UK; Biomolecular Mass Spectrometry and Proteomics Group, Bijvoet Center for Biomolecular Research, Utrecht Institute for Pharmaceutical Sciences, Utrecht University, Utrecht, The Netherlands; Netherlands Proteomics Center, Utrecht, The Netherlands; Hubrecht Institute, Royal Netherlands Academy of Arts and Sciences (KNAW), University Medical Center Utrecht, Uppsalalaan 8, 3584 CT Utrecht, The Netherlands; Department of Cardiology, University Medical Center Utrecht, Heidelberglaan 100, 3584 CX Utrecht, The Netherlands

**Keywords:** SORBS2, Heart failure, Cardiomyocyte, Integrins, Extracellular matrix

## Abstract

**Aims:**

In this study, we aimed to uncover genes associated with stressed cardiomyocytes by combining single-cell transcriptomic data sets from failing cardiac tissue from both humans and mice.

**Methods and results:**

Our bioinformatic analysis identified *SORBS2* as conserved *NPPA*-correlated gene. Using mouse models and cardiac tissue from human heart failure patients, we demonstrated that *SORBS2* expression is consistently increased during pathological remodelling, correlates to disease severity, and is regulated by GATA4. By affinity purification mass spectrometry, we showed SORBS2 to interact with the integrin–cytoskeleton connections. Cardiomyocyte-specific genetic loss of *Sorbs2* in adult mice changed integrin interactions, indicated by the increased expression of several integrins and altered extracellular matrix components connecting to these integrins, leading to an exacerbated fibrotic response during pathological remodelling.

**Conclusion:**

*Sorbs2* is a cardiomyocyte-enriched gene that is increased during progression to heart failure in a GATA4-dependent manner and correlates to phenotypical hallmarks of cardiac failure. Our data indicate SORBS2 to function as a crucial regulator of integrin interactions and cardiac fibrosis.

## Graphical Abstract

**Graphical Abstract:**
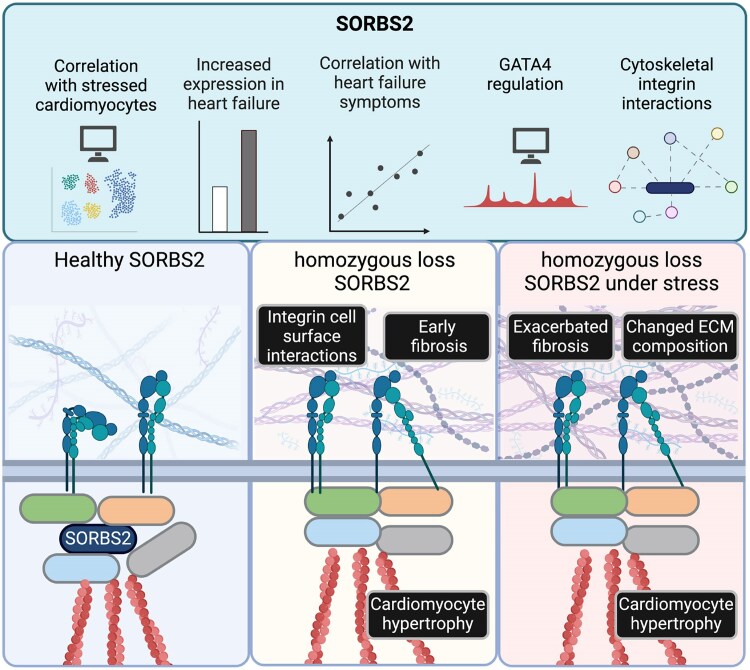

**Time of primary review: 48 days**

**See the editorial comment for this article ‘Cardiomyocyte-derived fibrosis as driver of cardiomyopathy’, by A. Martin-Garrido and J. Heineke, https://doi.org/10.1093/cvr/cvaf025.**

## 1. Introduction

Irrespective of the underlying aetiology, various cardiomyopathies can develop into a common chronic phase of functional cardiac impairment, which is known as heart failure.^1^ Heart failure is defined as a clinical syndrome in which patients exhibit insufficient cardiac output and/or increased intracardiac pressure caused by structural and/or functional cardiac abnormalities.^2^ While there have been substantial improvements in the prognosis for heart failure patients over the past decades, it continues to be associated with high mortality rates and impaired quality of life.^2^ Therefore, improving our understanding of fundamental aspects and identification of key molecules in the cardiac remodelling process remains of utmost importance.

Although different cardiac pathologies can underlie heart failure, they often share key aspects of pathological remodelling such as enlargement of cardiomyocytes (hypertrophy), re-expression of foetal genes such as *Nppa* and *Nppb* and fibrosis.^3–5^ Fibrosis is the excessive accumulation of (interstitial) extracellular matrix (ECM), which can lead to cardiac stiffening and as such results in a reduction in cardiac function.^3^ In addition, alterations in the ECM have a widespread effect on cells by influencing a variety of cellular processes such as survival, proliferation, or adhesion.^6^ As such, aberrations in the interaction of cardiomyocytes with their extracellular surrounding can itself induce fibrosis and cardiac failure,^7,8^ underscoring the importance of ECM–cardiomyocyte interplay.

Due to extensive recent technological advancements, single-cell transcriptomic atlas studies have provided an unprecedented wealth of valuable data uncovering gene expression profiles in all main cardiac cell types from both healthy and diseased cardiac tissue. By employing marker genes, one can zoom in into certain cell (sub)types and states such as those associated with disease or stress. Through the analysis of transcriptome-wide gene correlations, single-cell transcriptomic data sets can be leveraged to impartially identify novel genes important in cardiac disease. Differences between single-cell transcriptomic studies, such as applied technologies, use of species, and model, will inherently underlie differences in outcomes. Conversely, if a certain molecule or biological process is identified consistently regardless of such differences, one can anticipate its true biological importance.

To identify genes important in heart failure, we empowered the combination of independent single-cell transcriptomic data sets covering heart failure in both mice and humans^9,10^ and *Nppa* as anchor gene because of its well-established link with cardiomyocyte stress.^4,5^ Using this approach, we identified sorbin and SH3 Domain Containing 2 (*Sorbs2*) to be enriched specifically in stressed cardiomyocytes. SORBS2 belongs to a small family of adaptor proteins containing a sorbin homology domain and three SH3 domains, which are involved in cell adhesion, cytoskeletal organization, and growth factor signalling.^11^ While several recent studies showed SORBS2 to be induced during cardiomyopathies that can underlie heart failure,^12–14^ so far its molecular function and regulation in heart failure remain poorly understood. By using mouse models and cardiac tissue from human heart failure patients, we show that the expression of *Sorbs2* correlates to disease severity and is regulated by GATA4. By revealing the cardiac interactome of SORBS2, we identified SORBS2 to interact with numerous proteins connecting integrins to the cytoskeleton. These included the WAVE regulatory complex (WRC), shedding light on a protein complex not yet well characterized in cardiomyocyte biology. Genetic deletion of *Sorbs2* in adult cardiomyocytes led to aberrant integrin-related interactions and an exacerbation of the fibrotic response during pathological remodelling. Collectively, our data indicate that SORBS2 is essential for appropriate integrin function and thereby plays a pivotal role in cardiac fibrosis.

## 2. Methods

An extensive detailed description of all materials and methods is provided in the Supplementary material online, *Supplementary methods*.

### 2.1 Reanalysis of human and mouse cardiac single-cell sequencing data

Cell info and count data from *GSE109816*, *GSE121893*, and *GSE120064* were downloaded from the Gene Expression Omnibus (GEO). Data were analysed using R version 3.6.2 and Seurat v3.1.^15^ Filtering in line with the original publication was applied.^10^ Data were analysed using the Seurat SCTransform integration workflow.^15,16^ Log normalized values were used for differential expression and visualization of clusters.

### 2.2 Reanalysis of spatial transcriptomics

Spatial transcriptomic samples were downloaded from GSE214611 (see Supplementary material online, *Table S6*). Data were analysed using R version 4.1.3 and Seurat v4.0.1 and closely resembled the original publication.^17^ Different Visium slides were integrated using the Seurat integration workflow based on canonical correlation analysis.^15^ The FindAllMarkers function using the spatial data slot was applied to identify enriched genes for each cluster. These genes were used to classify clusters into remote, border, and infarct zone.

### 2.3 Experimental animals

All animal studies were performed in accordance with institutional guidelines and with approval of the Animal Welfare Committee of the Royal Netherlands Academy of Arts and Sciences. All animal experiments conform to the guidelines from Directive 2010/63/EU of the European Parliament on the protection of animals used for scientific purposes. For surgical interventions, mice were injected subcutaneously with the analgesic buprenorphine (0.05–0.1 mg/kg) at least 30 min before surgery. A second dose and third dose of buprenorphine (0.05–0.1 mg/kg) were given ∼8–12 and 24 hours post-surgery, respectively. Anaesthesia was induced by a mix of fentanyl (0.05 mg/kg), midazolam (5 mg/kg), and dexmedetomidine (0.125 mg/kg). If required, 1–2% isoflurane was added as maintenance anaesthesia. After the last echocardiographic measurement, animals were sacrificed by cervical dislocation under anaesthesia of 1–2% isoflurane. All mice used in the presented study were males of the C57Bl/6J background.

### 2.4 Human patient correlations

Patients with early-stage dilated cardiomyopathy (DCM) were included from the Maastricht Cardiomyopathy Registry.^18^ All individuals fulfilling the diagnosis of DCM, who underwent endomyocardial biopsies (EMBs) and had RNA-sequencing data available from their EMB, were included in the current study (*n* = 95). Details regarding RNA isolation, sequencing, and analysis are described in detail previously.^19^ All patients gave written informed consent, and the study was performed according to the declaration of Helsinki.

### 2.5 Uni- and multivariable regression analysis

For our human cohort, univariable linear regression analysis was performed to test the association between every individual clinical parameter (*n* = 42) and the *SORBS2* expression from the RNA sequencing data from the cardiac biopsy. Afterwards, all univariable clinical parameters that were significantly associated with *SORBS2* expression (*P* < 0.05) were included in a multivariable linear regression analysis, in which only clinical parameters were retained that had a *P*-value of <0.05. The same approach was taken to test association between clinical parameters and relative *Sorbs2* expression from quantitative real-time polymerase chain reaction (PCR) derived from our transverse aortic banding (TAB) timeline, in which a total of 17 individual clinical parameters were taken along in the univariable linear regression analysis. All statistical analyses were performed in R environment, version 4.0.4.

### 2.6 Western blot

Protein concentration of cardiac tissue was assessed using Bradford assay (Bio-Rad). Equal amounts of protein were loaded for SDS–PAGE and analysed by western blotting.

### 2.7 Quantitative real-time PCR

TRIzol reagent (Thermo Fisher, #15596018) was used to isolate total RNA from cells or heart tissue according to the manufacturer's instructions. After cDNA synthesis by reverse transcription, RT-qPCR was performed using iQ SYBR Green Supermix (Bio-Rad, #1708880). Primer sequences used for RT-qPCR are given in Supplementary material online, *Table S8*. Collagen expression was calculated as a combined score of *Col1a1*, *Col1a2*, and *Col3a1* expression (each variant determining 1/3th of the score), which comprise ∼90% of total the cardiac collagens.^20^

### 2.8 Immunohistochemistry and immunofluorescence

Immunohistochemistry and immunofluorescence were performed on paraffin-embedded cardiac tissue slices. Haematoxylin and eosin, Picro Sirius Red, and Alcian blue stainings were performed on whole heart slices that were imaged using a slide scanner (Olympus, VS200). Whole ventricular fibrosis (based on positivity for Picro Sirius Red) and positivity for Alcian blue were determined using QuPath.^21^*In situ* cell death detection kit (Roche, #12156792910) was used for TUNEL labelling according to the manufacturer's instructions.

### 2.9 Affinity purification mass spectrometry

Left ventricular tissue was lysed after which equal amounts of protein were used for immunoprecipitation using Magnetic Dynabeads Protein G (Thermo Fisher, #10003D) coupled to a SORBS2 (Sigma-Aldrich, #SAB4200183) or WAVE2 (Cell Signaling, #3659) antibody or isotype control antibody (Thermo Fisher, #14-4714-85 or #02-6102). Immunoprecipitated protein samples were further subjected to SDS–PAGE, digestion and subsequent resuspended peptides were used for mass spectrometry. Protein identification was performed using MaxQuant 1.6.17.0.,^22^ and after identification of 48 significant interaction partners, these were uploaded to the STRING database for gene ontology (GO) and network analysis.^23^

### 2.10 RNA-sequencing

Library preparation, sequencing, pre-processing of RNA-sequencing data, and further details for analysis are described in the Supplementary material online, *Supplementary methods*. Raw counts were analysed using R version 4.1.3 and DESeq2 version 1.32.0.^24^ We used the GSEA-MSigDB application to imply biological processes based on geometric normalized count tables derived from DESeq2.^25^

### 2.11 hiPS-CM culture

Human iPS-cells were obtained from ATCC (#ACS-1026). Differentiation into human-induced pluripotent stem cell-derived cardiomyocytes (hiPS-CMs), purity assessment, and immunoprecipitation experiments using hiPS-CMs are described in the Supplementary material online, *Supplementary methods*. hIPS-CMs were treated with 10 nM of control siRNA (Thermo Fisher, #439084) or a siRNA targeting *Gata4* (Thermo Fisher, #4392420, ID: s535120) for 72 hours. siRNA transductions were performed using Opti-MEM (Gibco™, #11058021) and Lipofectamine™ RNAiMAX (Thermo Fisher, #13778075). Endothelin-1 (ET-1) (Sigma-Aldrich, #E7764) or vehicle control (DMSO) treatment occurred at a concentration of 10 nM 48 hours and again at 24 hours before collection.

### 2.12 Promotor activity assay

*Sorbs2* promotor activity was determined using the Dual Luciferase Reporter® Assay System (Promega #E1910) and a Luminometer (Berthold Technologies, Centro XS^3^ LB 960). A 451 bp region (mm39 chr8:46080750-46081200) covering GATA4 chromatin immunoprecipitation sequencing (ChIP-seq) peak at *Sorbs2* TSS-2 was used to test promotor activity.

### 2.13 Statistics

Statistical analysis of affinity purification mass spectrometry, RNA-sequencing, and reanalysis of ChIP-seq data is described in detail in the Supplementary material online, *Supplementary methods*. All remaining statistical analyses were performed using Prism version 9.5.1. Student's and nested *t*-tests, as well as analyses of variance (ANOVAs), were conducted after confirming that the necessary assumptions, such as normality and homoscedasticity, were met. In cases where these assumptions were not directly fulfilled, tests were applied to transformed data. If transformation did not suffice to meet the required assumptions, non-parametric tests were employed. In a few instances involving two-way ANOVA analyses, the prerequisites could not be met, and this is mentioned specifically in the corresponding legend. Each statistical test or multiple comparison correction is mentioned in the corresponding figure and table legend.

### 2.14 Data and material availability

RNA-sequencing raw and processed data are deposited in the GEO repository and available under accession code: GSE243463. All data needed to evaluate the conclusions in the paper are present in the paper and/or the Supplementary material.

## 3. Results

### 3.1 Single-cell transcriptomic analysis identifies genes involved in cardiomyocyte failure

**Figure 1:**
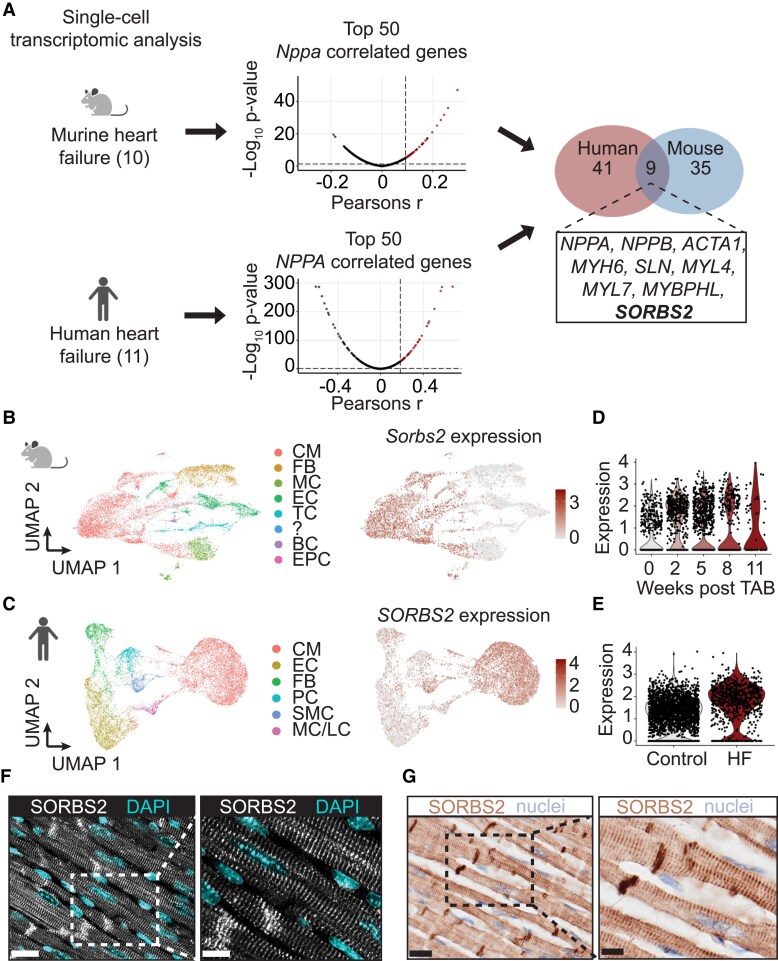
Single-cell transcriptomics identifies *Sorbs2* as *Nppa*-correlated gene in mice and human heart failure. (*A*) Schematic overview of the pipeline to identify top-ranked conserved *NPPA*-correlated genes in heart failure. Of the top 50 mouse *Nppa*-correlated genes, 44 had a known human homolog. (*B*, *C*) UMAP plot for all cells [*n* = 11492 (*B*) or *n* = 12 861 (*C*)] present in murine (*B*) and human (*C*) data set in which cells are colour coded by type (left panel) or by *Sorbs2* expression (right panel). (*D*) Violin plot of *Sorbs2* expression in CMs at different time points after TAB. (*E*) Violin plot of *SORBS2* expression in CM from control or failing (HF) hearts. (*F, G*) SORBS2 localization in mouse (*F*) and human (*G*) heart tissue by immunostaining. Scale bar: 20 μm (left panel) and 10 μm (right panel). BC, B cell; CM, cardiomyocyte; EC, endothelial cell; EPC, epicardial cell; FB, fibroblast; LC, lymphocyte; MC, monocyte; PC, pericyte; SMC, smooth muscle cell; TC, T cell.

In an effort to improve our understanding of cardiac failure, we aimed to determine gene expression changes occurring specifically in stressed cardiomyocytes of both mice and human. *Nppa* and *Nppb*, cardiac genes that encode for atrial natriuretic peptide and brain natriuretic peptide, respectively, are predominantly expressed in the atria in the adult heart. Ventricular expression occurs during the embryonic and foetal stages and is strongly induced again under conditions of stress, with *Nppa* exhibiting the strongest stress response of the two.^4,5^ Because of the tight connection between *Nppa* expression and cardiomyocyte stress,^4,5^ we interrogated single-cell transcriptomic data sets from failing hearts^9,10^ to find genes that are robustly correlated to *Nppa* expression in both murine and human cardiomyocytes (*Figure 1A*; see Supplementary material online, *Figure S1*). To this end, we employed a rank-based comparison approach to identify the genes for which the expression most strongly correlated with *Nppa*. Our analysis revealed nine genes that were overlappingly up-regulated in stressed cardiomyocytes from both mouse and human (*Figure 1A*). All these genes appeared to be predominantly expressed in cardiomyocytes (see Supplementary material online, *Figure S2A* and *B*) and induced in response to cardiac stress in both human and mouse (see Supplementary material online, *Figure S2C* and *D*). Among these nine genes, we recognized classical marker genes of cardiomyocyte stress, such as *NPPB* and α-skeletal muscle actin (*ACTA1*).^26,27^*ACTA1* exhibits elevated expression in various instances of cardiomyocyte stress across multiple species.^26–28^*ACTA1* also has a clear link to mechanical stress that manifests for example as induced expression in cardiomyocytes in the periphery of fibrotic regions.^26,27^ In this list, we also identified genes less well known for their role in cardiomyocyte failure such as Myosin Light Chain 4 and 7 *(MYL4* and *MYL7*), myosin binding protein H-like (*MYBPHL*), and sarcolipin (*SLN*). *MYL4*,^29^*MYL7*,^29^*SLN*,^30^ and *MYBPHL*^31^ exhibit similar to *NPPA*, predominant atrial expression in the adult heart. Likewise to the *NPPA* stress response, increased ventricular expression of *MYL4* and *SLN* has been observed during cardiac disease.^29,32,33^ Surprisingly, our analysis additionally identified myosin heavy chain 6 (*MYH6*), while the other myosin heavy chain isoform *MYH7* is the isoform known to be induced upon stress. Also, *MYH6* has been observed to show atrial preference, depending on the species and developmental stage.^29^ This shared aspect between *NPPA* and *MYH6*, and conceivably *MYL4*, *MYL7*, *MYBPHL*, and *SLN*, suggests a degree of regulatory overlap that likely underlies the presence in our final list.

In addition to known markers of cardiomyocyte stress and failure, we also identified the expression of *SORBS2* to correlate to the expression of *NPPA*. SORBS2 belongs to a small family of adaptor proteins having a sorbin homology domain and three SH3 domains, which are known to regulate cell adhesion, cytoskeletal organization, and growth factor signalling.^11^ Similar to ACTA1, SORBS2 expression does not exhibit atrial preference.^34^ While *SORBS2* has recently been linked to various cardiomyopathies,^12–14,35^ its biological function and regulation during cardiac remodelling remain largely unknown.

Uniform Manifold Approximation and Projection (UMAP) projection of the single-cell transcriptomic analysis indicated *SORBS2* to be highly enriched in cardiomyocytes from the mouse and human heart compared with other cardiac cell types (*Figure 1B* and *C*). As expected, based on the correlation with *Nppa*, we found the expression of *Sorbs2* to be increased in cardiomyocytes after TAB in mice (*Figure 1D*). Also, in cardiomyocytes of failing human hearts, the expression of *SORBS2* was increased compared with the levels detected in control cardiomyocytes (*Figure 1E*). Although smooth muscle cells also show *SORBS2* expression, the increased expression is restricted to cardiomyocytes (see Supplementary material online, *Figure S3A* and *B*). Histological assessment of mouse and human heart sections showed a clear striated pattern of SORBS2 throughout cardiomyocytes and increased abundance at the intercalated disc (*Figure 1F* and *G*; Supplementary material online, *Figure S4A* and *B*). The observed localization pattern did not appear to be influenced by stress as shown by histological assessment of sections from hypertrophic cardiomyopathy, ischaemic cardiomyopathy, or TAB exposed hearts (i.e. hypertension) (see Supplementary material online, *Figure S4C*–*E*). Together, these data support the value of using single-cell sequencing to identify genes relevant for cardiomyocyte failure to unveil new biology and indicate SORBS2 to be increased in stressed cardiomyocytes.

### 3.2 *Sorbs2* expression correlates to cardiac function and remodelling in murine and human heart failure

**Figure 2:**
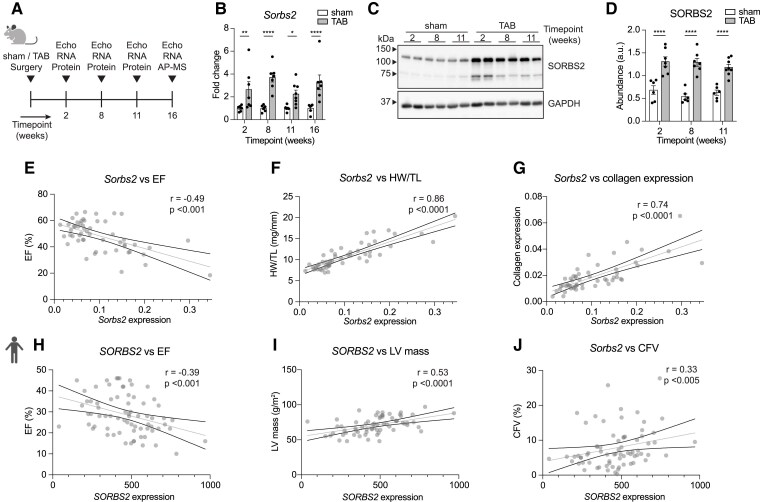
*Sorbs2* expression increases during pathological remodelling and correlates to cardiac function and remodelling. (*A*) Schematic overview of the experimental set-up (*Figure 4*). (*B*) *Sorbs2* mRNA expression by RT-qPCR (*n* = 6–8). (*C*, *D*) SORBS2 protein expression by western blot (representative image in *C*) quantified in *D* (*n* = 6–8). (*E–G*) Correlation analysis of murine *Sorbs2* mRNA expression by RT-qPCR and EF, HW/TL ratio, and collagen expression (*n* = 53). (*H–J*) Correlation analysis of human *SORBS2* mRNA expression (normalized read count) and EF, LV mass, and CFV (*n* = 67–72). Correlations in *E*–*J* were determined by Spearman's *r* and *P*-values were tested in a two-tailed manner. Grey solid lines are the best fit lines from a simple linear regression, and black solid lines indicate 95% confidence interval of the best fit. Significance tested by a two-way ANOVA with Šidák's multiple comparisons test for condition comparison at each time point (*B*, *D*). Error bars: SEM. Dots represent biological replicates. Significance levels: *P < 0.05, **P < 0.01, and ****P < 0.0001. a.u., arbitrary unit; AP-MS, affinity purification mass spectrometry; CFV, collagen fractional volume; Echo, echocardiography; EF, ejection fraction; HW/TL, heart weight to tibia length ratio; LV mass, left ventricular mass; TAB, transverse aortic banding.

To start exploring the function of SORBS2 during heart failure in more detail, we subjected mice to TAB or sham surgery and analysed the hearts 2, 8, 11, and 16 weeks later (*Figure 2A*). We found *Sorbs2* expression to be consistently increased at both messenger RNA (mRNA) and protein level, at all time points analysed post-TAB (*Figure* 2B–D; see Supplementary material online, *Figure S5*). SORBS2 exhibits different isoforms that represent as bands between ∼70 and 140 kDa in the heart,^13^ which all display increased abundance (*Figure 2C*). As mentioned before, hypertrophy and fibrosis are key features of pathological remodelling and are associated with deterioration of cardiac function and ultimately failure.^3^ Echocardiographic measurements showed that the increase in *Sorbs2* expression after TAB correlated to a reduction in cardiac function as assessed by ejection fraction (EF) (*Figure 2E*; see Supplementary material online, *Table S1*). Furthermore, we observed that the increased *Sorbs2* expression correlates to cardiac hypertrophy as measured by the heart weight to tibia length (HW/TL) ratio and collagen expression, serving as a surrogate of fibrosis (*Figure 2F* and *G*). To determine whether these correlations are also conserved in the human failing heart, we made use of a cohort of patients that were diagnosed with early-stage heart failure and underwent EMBs for RNA-sequencing in addition to a plethora of clinical assessments.^19^ Also, in this human cohort, we could demonstrate that the expression of *SORBS2* correlated to impaired cardiac function and levels of hypertrophy and fibrosis, as assessed by EF, left ventricular mass (LV mass), and collagen fractional volume (CFV) in the EMB, respectively (*Figure 2H–J*). Multivariable linear regression analysis, to explore which clinical variables are independently associated with *SORBS2* expression, indicated positive associations of *SORBS2* expression with cardiac fibrosis, hypertrophy, and duration of symptoms in human heart failure patients (see Supplementary material online, *Table S2*). Similar to the human cohort, multivariable linear regression analysis in mice indicated positive associations between *Sorbs2* expression and collagen expression and measures related to hypertrophy such as HW/TL and left ventricular posterior wall thickness (see Supplementary material online, *Table S3*). In summary, these findings show that *Sorbs2* expression increases in cardiomyocytes in response to stress, and this correlates to cardiac function and remodelling in mice and human heart failure patients.

### 3.3 GATA4 is a transcriptional regulator of *Sorbs2*

**Figure 3:**
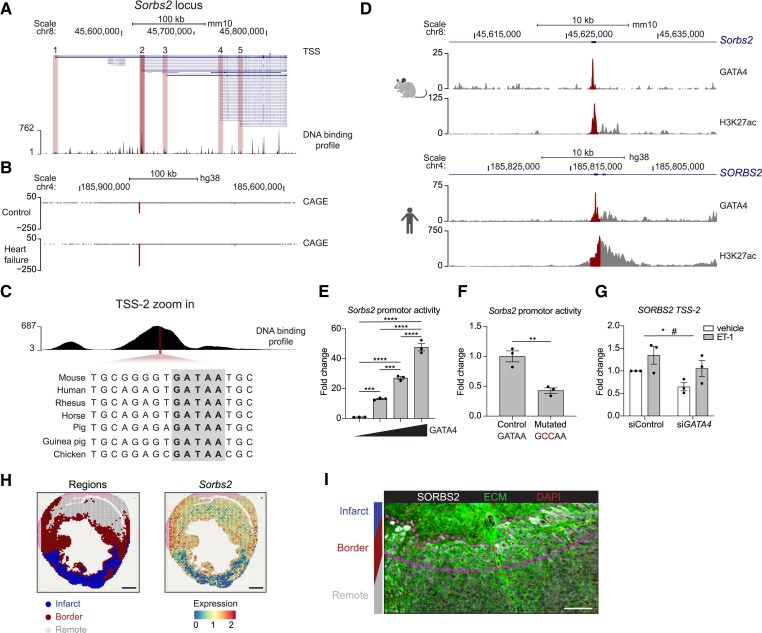
GATA4 regulates *Sorbs2* expression at a stress-sensitive promotor region. (*A*) Schematic overview of the *Sorbs2* locus in the mouse reference genome and density plot of DNA binding at this region derived from the ReMap atlas. Transcript track: NCBI RefSeq updated annotation release 108.20200622. Genomic regions around a TSS are marked in red, and TSS-2 is marked in dark red. (*B*) Coverage plot of TSS activity from CAGE in the orthologous human region shown in A. Positive and negative coverage indicate reads from plus and minus strand, respectively. Dark red signal marks TSS-2 region. (*C*) A zoom-in of the dark red marked region in A, indicating the location of a conserved GATA4 motif. (*D*) GATA4 and H3K27ac ChIP-sequencing data of the mouse heart (upper panels) and human cardiomyocytes (lower panels) with TSS-2 region marked in dark red. *Y*-axis: -log_10_(*P*-value). (*E*) Promotor activity of TSS-2 with increasing quantities of GATA4 expression plasmid. An ordinary one-way ANOVA with Tukey's multiple comparisons test was applied (*n* = 3; biological replicates). (*F*) Promotor activity of TSS-2 after mutating the GATA4 binding site indicated in panel C. An unpaired student's *t*-test (two-sided) was applied (*n* = 3; biological replicates). (*G*) *SORBS2* mRNA expression measured by RT-qPCR after a combined treatment of siRNA targeting *GATA4* or non-targeting control and ET-1 or vehicle. Ordinary two-way ANOVA indicates main effect for si*GATA4*(*) and ET-1(#) treatment (*n* = 3; independent differentiation). (*H*) Spatial transcriptomic reanalysis of the mouse heart 7 days post myocardial infarction (MI). Scale bar: 1 mm. (*I*) SORBS2 expression surrounding border zone area (within dashed pink lines) 7 days post-MI by immunostaining. ECM: extracellular matrix. Scale bar: 100 μm. Error bars: SEM. Dots represent biological replicates. Significance levels: #*P* < 0.05,*P < 0.05, **P < 0.01, ***P < 0.001, ****P < 0.0001. CAGE, cap analysis of gene expression; ET-1, endothelin-1; TSS, transcription start site.

To better understand the transcriptional regulation of *SORBS2*, we next investigated its genomic organization. The *Sorbs2* locus is highly complex as differences in transcription start sites (TSSs) and alternative splicing result in tens of transcript variants depending on genome annotation version and species (*Figure 3A*; Supplementary material online, *Figure S6A*). To gain more insight into the stress-induced expression of *Sorbs2*, we assessed potential regulatory elements within the *Sorbs2* locus using a large-scale integration of DNA-binding experiments including over 5500 and 8000 experiments for mouse and human, respectively.^36^ We aligned this integrated DNA-binding profile with the different potential TSS of *Sorbs2* and found a high enrichment of DNA binding at TSS-2, indicative of a regulatory site at this genomic region (*Figure 3A*). Using primers designed to uniquely detect the different TSS regions, we analysed the mRNA expression of transcripts originating from these different TSSs in cardiac tissue of our TAB time course experiment. This showed that only transcripts originating from TSS-2 were consistently up-regulated after TAB-induced pathological remodelling (see Supplementary material online, *Figure S6A* and *B*). To validate our findings, we turned to an atlas of human cardiac promotors generated using cap analysis of gene expression (CAGE).^37^ This method utilizes a 5′ cap-trap technique to enable sensitive quantification of gene expression from promotors. This analysis confirmed TSS-2 to be the main start region and showed a robust increased expression in human heart failure (*Figure 3B*). We further assessed chromatin accessibility of TSS-2 by exploring a human tissue-based single-cell atlas of Assay for Transposase-Accessible Chromatin using sequencing.^38^ This showed that from all cardiac cell types present in this atlas, chromatin accessibility at TSS-2 was clearly enriched in cardiomyocytes (see Supplementary material online, *Figure S7*).

To identify transcriptional regulators of *Sorbs2*, we analysed the genomic region surrounding TSS-2 in more detail and noticed a highly conserved GATA4 motif (*Figure 3C*). GATA4 is a well-known transcriptional regulator of cardiac development and disease, and its transcriptional activity in the heart is known to be induced after mechanical stress.^39–41^ We found *Sorbs2* to be correlated with *Nppa*, which is also under transcriptional control of GATA4,^41^ suggesting a possible regulatory role of GATA4 on *Sorbs2* expression. Making use of available GATA4 and H3K27ac ChIP-sequencing data of the mouse heart and human cardiomyocytes,^41,42^ we were able to confirm the binding of GATA4 at the identified promotor region and its transcriptional activity (*Figure 3D*). To substantiate that GATA4 regulates *Sorbs2* expression, we cloned the identified promotor region surrounding TSS-2 into a luciferase reporter construct and performed luciferase assays. These data demonstrated that GATA4 can dose-dependently activate the identified *Sorbs2* promotor region (*Figure 3E*). Mutagenesis of the GATA4-binding motif introduced a significant reduction in luciferase activity, indicating GATA4 indeed to be acting through the identified recognition sequence (*Figure 3F*). Moreover, *Gata4* knockdown or GATA4 stimulation by its known activator ET-1^43^ in human-induced pluripotent stem cell-derived cardiomyocytes (hiPS-CMs) resulted in a decrease and increase in *Sorbs2* expression from TSS-2, respectively, further supporting the regulatory influence of GATA4 on SORBS2 expression (*Figure 3G*).

Cardiac remodelling in response to ischaemic injury is known to cause the formation of a rigid scar, posing a high mechanical strain on neighbouring cardiomyocytes. As GATA4 transcriptional activity increases upon mechanical stress,^39,40^ we used available spatial transcriptomic data of mice hearts after myocardial infarction to see if *Sorbs2* expression would be induced in the periphery of fibrotic regions^17^ (see Supplementary material online, *Figure S8*). This showed a clear elevation of *Sorbs2* expression in the neighbouring region of the scar (*Figure 3H*). We could confirm this local stress-induced elevation by immunohistochemistry for SORBS2, which also demonstrated an increase in SORBS2 expression in cardiomyocytes directly flanking the fibrotic scar (*Figure 3I*). In summary, we identified the primary promotor region of *Sorbs2*, which is GATA4 sensitive and contributes to the induced *Sorbs2* expression in heart failure.

### 3.4 Identification of SORBS2-binding partners indicates a role in cytoskeletal–integrin interactions

**Figure 4:**
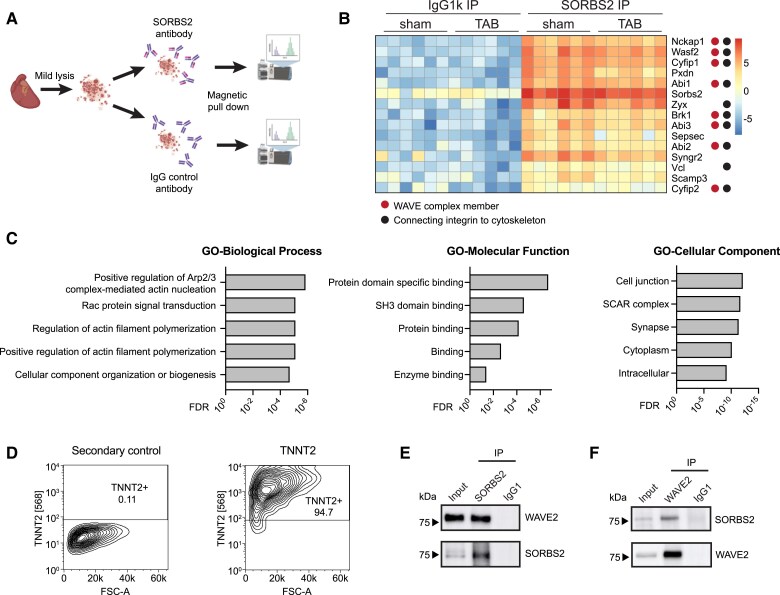
SORBS2 binds proteins connecting integrins to the cytoskeleton. (*A*) Schematic overview of the experimental set-up. (*B*) Heatmap of the top 15 SORBS2 interaction partners, ordered based on significance from top to bottom. Colour scale: abundance based on normalized intensity-based absolute quantification (iBAQ) values. Different columns represent biological replicates (*n* = 6 per condition). (*C*) GO results using all significant SORBS2 interaction partners. FDR values are based on Benjamini–Hochberg procedure. (*D*) Contour plot from fluorescence-activated cell sorting analysis of hiPS-CMs purity used for co-immunoprecipitation in *F*. Percentage purity is indicated as number in of cells positive for cardiac troponin T (TNNT2) in both plots. (*E*) Co-immunoprecipitation of WAVE2 using SORBS2 as bait antibody in hiPS-CMs. (*F*) Co-immunoprecipitation of SORBS2 using WAVE2 as bait antibody on cardiac tissue lysate. FDR, false discovery rate; FSC-A: forward scatter area; GO, gene ontology; TAB, transverse aortic banding.

As SORBS2 is an adaptor protein, its function is likely to be regulated by the proteins it binds to. To examine its functional role in the relevant environment, we set out to identify the *in vivo* cardiac-binding partners of SORBS2 by performing affinity purification mass spectrometry in healthy and diseased mouse hearts (*Figure 4A*). Using several biological replicates and after stringent filtering, we were able to identify 48 significant cardiac-binding partners of SORBS2 (see Supplementary material online, *Figure S9* and *Table S4*). We found little differential binding of SORBS2 between healthy and TAB hearts, indicating that the function of SORBS2 in heart failure is likely attributed to differential expression rather than differential protein binding (*Figure 4B*; Supplementary material online, *Table S4*). Our data indicated that SORBS2 predominantly binds to proteins that are important for connecting integrins to the actin cytoskeleton, which matches its localization pattern (*Figure 4B*). Integrins are transmembrane receptors forming the connection between the cytoskeleton and the ECM. The extracellular domain of integrins binds to the ECM and other cells while the cytoplasmic tail is bridged to the actin cytoskeleton by a layer of adaptor proteins. Together, these protein assemblies that connect the actin–cytoskeleton to the ECM are commonly known as focal adhesions or costameres in case of cardiomyocytes.^44^ These SORBS2-binding proteins suggest that SORBS2 affects the ECM–cytoskeletal connection. In the SORBS2 pull-down, we found a strong over-representation of proteins that are part of the WRC (*Figure 4B*). The WRC is known to regulate cytoskeletal actin polymerization and as such is involved in the connection between the actin cytoskeleton and integrins.^45,46^ GO analysis of the SORBS2 interactome revealed terms related to its location at cell junctions, regulation of the actin cytoskeleton, and its binding function as adaptor protein (*Figure 4C*).

As SORBS2 is highly enriched in cardiomyocytes, it is interesting to note that the WRC is exceptionally ill studied in cardiomyocytes. To ensure that the interaction between SORBS2 and the WRC indeed occurred in cardiomyocytes, we performed co-immunoprecipitation experiments on endogenous SORBS2 protein in a highly pure population of hiPS-CM (*Figure 4D*). Western blot analysis confirmed that SORBS2 and the WRC bind in cardiomyocytes and additionally demonstrated conservation of the interaction in a human model (*Figure 4E*). We further validated the binding of SORBS2 and the WRC in the heart by a reversed pull-down using a bait antibody against WAVE2, a protein member of the WRC (*Figure 4F*).

Integrins are involved in a plethora of processes, such as adhesion, hypertrophy, and fibrosis.^8,44,47^ The data presented above show that SORBS2 predominantly binds to proteins important in the connection between integrins and the actin–cytoskeleton. Given the known association of SORBS2 with cell adhesion^35^ and our observed associations with hypertrophy and fibrosis, these results support an intertwined involvement of SORBS2 and integrin-mediated processes in cardiac remodelling. Taken together, we provide the *in vivo* cardiac interactome of SORBS2 and show that SORBS2 binds to proteins that are involved in the linkage of cytoskeletal actin filaments to integrins and reveal the WRC as not yet well-characterized protein complex in cardiomyocyte biology.

### 3.5 *Sorbs2* deletion exacerbates the cardiac fibrotic response under stress conditions

**Figure 5:**
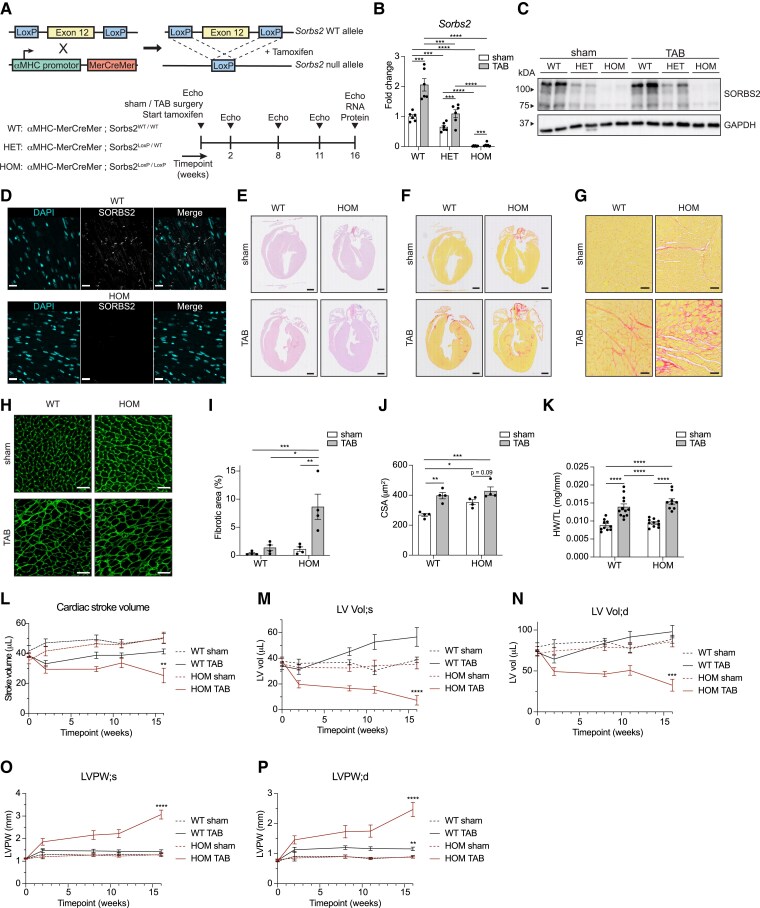
Cardiomyocyte specific loss of *Sorbs2* in the adult heart exacerbates fibrosis. (*A*) Schematic overview of the experimental set-up. (*B*) mRNA expression of *Sorbs2* (all variants) by RT-qPCR (*n* = 6 per condition). (*C*) Protein expression of SORBS2 by western blot. (*D*) Representative images of SORBS2 staining in WT hearts and after *Sorbs2* loss. Scale bar: 20 μm. (*E*, *F*) Cardiac morphology and fibrosis showed by representative images of haematoxylin and eosin staining (*E*) and Picro Sirius red staining (*F*). Scale bars: 1 mm. (*G*) Zoomed-in images corresponding to *F*. Scale bars: 50 μm. (*H*) Wheat germ agglutinin (WGA) staining (*H*) used for cardiomyocyte CSA quantification shown in *J*. Scale bars: 50 μm. (*I*) Quantification of ventricular fibrosis in *F* (*n* = 4). (*J*) Quantified cardiomyocyte CSA. Between 543 and 1454 cells per heart were quantified (*n* = 4 hearts). (*K*) Cardiac weight measured by HW/TL ratio (*n* = 9–12). (*L–P*) Cardiac stroke volume (*L*), left ventricular end volume at systole (*M*) and diastole (*N*), and left ventricular posterior wall thickness at systole (*O*) and diastole (*P*). *n* = 7–14 biological replicates per condition per time point. Significance was determined by a two-way ANOVA and Tukey's multiple comparisons test at 16-week time point (*B*, *I*–*N*). For *L*–*N*, asterisks are true for comparison with every other condition. Error bars: SEM. Dots represent biological replicates. Significance levels: *P < 0.05, **P < 0.01, ***P < 0.001, and ****P < 0.0001. CSA, cardiomyocyte cross sectional area; Echo, echocardiography; HET, *Sorbs2^+/−^*; HOM, *Sorbs2^−/−^*; TAB, transverse aortic banding; WT, wild type.

To further delineate the molecular function of SORBS2 and assess its therapeutical potential, we studied the effect of *Sorbs2* loss in the healthy and diseased adult mouse heart. So far, previous studies showed that the loss of *Sorbs2* can lead to a phenotype resembling arrhythmogenic right ventricular cardiomyopathy (ARVC),^12^ DCM,^13^ and congenital heart disease.^35^ These studies used continuous loss of function models that are in particular valuable to study the effect of *Sorbs2* loss during development or to mimic loss of function mutations. In light of the robust up-regulation of *Sorbs2* expression specifically after injury, we generated cardiomyocyte-specific inducible *Sorbs2* heterozygous (*Sorbs2*^+/−^) and homozygous knockout mice (*Sorbs2*^−/−^) (*Figure 5A–D*). To this purpose, we crossed mice harbouring a tamoxifen-inducible Cre recombinase under control of the alpha-myosin heavy chain promotor (αMHC-MerCreMer) with mice containing LoxP sites surrounding a critical exon of *Sorbs2* leading to a loss of SORBS2 in case of removal.^48^ Adult mice were subjected to TAB or sham surgery and injected intraperitoneally with tamoxifen on 3 consecutive days and followed up for 4 months post-injection. All control mice were on an αMHC-MerCreMer background and received tamoxifen injections.

Although *Sorbs2*^+/−^ mice displayed an approximate normalization of *Sorbs2* expression after TAB-induced pathological remodelling (*Figure 5B* and *C*), this did not influence cardiac function or remodelling post-TAB (see Supplementary material online, *Figure S10A*–*I*). Also, at a molecular level, the heterozygous loss of *Sorbs2* did not affect cardiac stress as indicated by gene expression of common stress markers (see Supplementary material online, *Figure S10J*). These data suggest that merely blocking the up-regulation of *Sorbs2* has neither a protective nor harmful effect. However, already under baseline conditions, *Sorbs2*^−/−^ mice displayed a trending increase in fibrosis and an increase in cardiomyocyte hypertrophy (*Figure 5F–K*; Supplementary material online, *Table S5*). In response to stress, the fibrotic response became significantly worse in the absence of *Sorbs2* (*Figure 5G* and *I*). Besides a slight increase in cardiomyocyte size, no gross morphological or functional changes were observed at this time point when we compared *Sorbs2*^−/−^ with wild-type mice (*Figure 5E* and *I–N*). On the other hand, the loss of *Sorbs2* during TAB-induced pathological remodelling proved to be detrimental as *Sorbs2*^−/−^ mice displayed significantly more cardiac fibrosis and concentric hypertrophy resulting in highly reduced left ventricular volume and cardiac stroke volume and increased posterior wall thickness (*Figure 5L–P*). To test if the fibrosis was not a merely replacement of potential cell death as a consequence of the combined stress from TAB induces pathological remodelling and loss of *Sorbs2*, we also assessed cell death (see Supplementary material online, *Figure S11A*). This showed no significant differences due to the loss of *Sorbs2* and TAB when compared with TAB hearts of wild-type mice, which further confirms an induced fibrotic response due to the loss of *Sorbs2* (see Supplementary material online, *Figure S11B*). These data demonstrate that the loss of *Sorbs2* in adulthood causes a fibrotic phenotype, which becomes excessive in a disease setting.

### 3.6 SORBS2 affects integrin interactions and ECM composition

**Figure 6:**
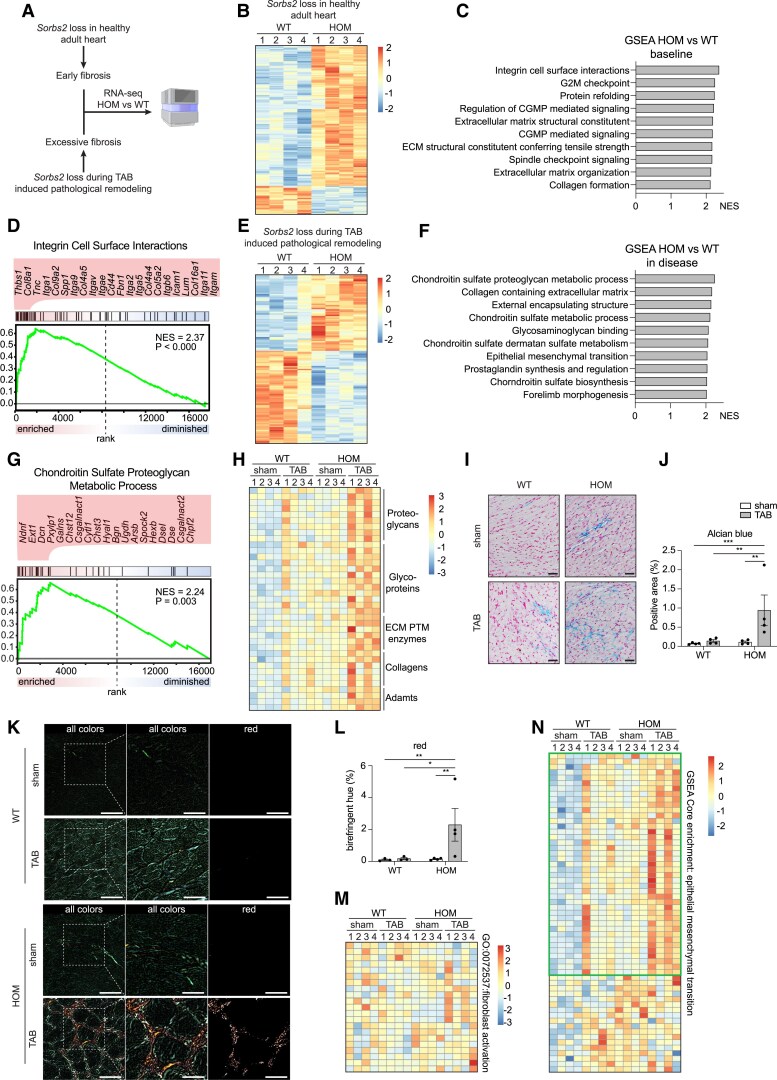
RNA-sequencing indicates SORBS2 as regulator of integrin interactions and fibrosis. (*A*) Schematic overview of the experimental set-up. (*B*) Heatmap of significant differential expressed genes with log_2_(fold change) > 0.5, between HOM and WT mice in healthy setting. Colour scale: *z*-scores. (*C*) Top 10 terms based on NES resulting from GSEA upon comparison of HOM and WT mice in baseline setting. (*D*) Enrichment plot of the highest scoring term in *C*. The most enriched corresponding genes are indicated and rank-ordered from left to right. (*E*) Heatmap of significant differential expressed genes with log_2_(fold change) > 0.5, between HOM and WT mice after TAB. Colour scale: *z*-scores. (*F*) Top 10 terms based on NES resulting from GSEA upon comparison of HOM and WT mice 16 weeks after TAB. (*G*) Enrichment plot of the highest scoring term in *F*. The most enriched corresponding genes are indicated and rank ordered from left to right. (*H*) Heatmap of a gene selection underlying term in *F*. Colour scale: *z*-scores. (*I*, *J*) Representative images of Alcian blue staining (*I*), quantified in *J* (*n* = 4). Scale bars: 50 μm. (*K–L*) Representative images of polarized light microscopy images (*K*), red birefringent hue quantified in *L*. Scale bars: 5 μm (left panel) and 2.5 μm (middle and right panel). Quantification shown in right panel (*n* = 3–4). (*M*) Heatmap of GO term 0072537: fibroblast activation. Colour scale: *z*-scores. (*N*) Heatmap epithelial–mesenchymal transition from GSEA analysis. Colour scale: *z*-scores. Green box indicates genes with similar pattern as fibrotic phenotype. Error bars: SEM. Dots represent biological replicates. Significance determined by a two-way ANOVA with Tukey's multiple comparisons test. **P < 0.01 and ***P < 0.001. Numbers above heatmaps indicate biological replicates. ECM PTM, extracellular matrix post-translational modification; GSEA, gene set enrichment analysis; HOM, *Sorbs2^−/−^*; NES, normalized enrichment score; TAB, transverse aortic banding; WT, wild type.

To assess the molecular differences underlying the fibrotic response due to loss of *Sorbs2*, we performed RNA-sequencing on the hearts of wild-type and *Sorbs2*^−/−^ mice under basal and disease conditions (*Figure 6A*). In line with the fibrotic phenotype and our protein interaction partner data, the transcriptomic programme affected by loss of *Sorbs2* in the adult healthy heart was mainly related to integrin interactions and ECM changes as indicated by gene set enrichment analysis (GSEA) (*Figure 6B–D*; Supplementary material online, *Figure S12A* and *B*). Underlying these GSEA terms were genes encoding for integrins, collagens, and several other extracellular proteins such as *Thbs-1* or *Tnc*, which are glycoproteins that play a role in cell-ECM interactions (*Figure 6D*; Supplementary material online, *Figure S12B*). The induction of these genes could be confirmed by RT-qPCR in a larger sample size (*n* = 6) (see Supplementary material online, *Figure S12C*). Consistent with the severe fibrotic phenotype of *Sorbs2*^−/−^ mice post-TAB, GSEA after banding primarily revealed changes related to ECM composition and organization when compared with wild-type mice (*Figure 6E–G*; Supplementary material online, *Figure S12D* and *E*). Underlying these terms were largely genes encoding for proteoglycans, glycoproteins, certain collagens, or ECM post-translational modification enzymes such as *P3h2*, *Col4a5*, or *Muc1* which we also confirmed to be up-regulated by RT-qPCR in a larger sample size (*n* = 6) (see Supplementary material online, *Figure S12F*). Although not directly related to the top GSEA terms, among the most significant up-regulated genes, we also identified *Nmrk2* that is an integrin beta-1-binding protein^49^ and *Dok5* that is involved in pro-fibrotic signalling together with *Igfbp5*,^50^ that we also validated to be up-regulated in a larger sample set (*n* = 6) (see Supplementary material online, *Figure S12F*). Whereas the GSEA terms in baseline conditions indicated a somewhat broader range of cellular processes affected by the loss of *Sorbs2*, these were more restricted to ECM remodelling processes after TAB (*Figure 6C* and *F*). This underscores that the fibrotic response is the primary aspect changed due to loss of *Sorbs2* in adult cardiomyocytes post-TAB compared with wild-type mice. Heatmap projection of our RNA-sequencing results corroborated the presence of alterations in genes related to ECM remodelling in *Sorbs2*^−/−^ mice under baseline conditions, with a more advanced induction in combination with TAB-induced stress (*Figure 6H*). In line with our RNA-sequencing data, we found a clear increase in glycoproteins and/or proteoglycans in the *Sorbs2*^−/−^ mice as shown by Alcian blue staining (*Figure 6I* and *J*). To further study the changes in ECM composition, we employed polarized light microscopy, a technique capable of identifying the maturity and cross-linking of collagen fibres. Whereas immature collagen fibres are detected in the green colour hue range, more matured and cross-linked collagen fibres are detected in the red colour hue range.^51^ This analysis showed an increased percentage of collagen fibres that appeared in the red hue range in *Sorbs2*^−/−^ mice after TAB, which suggests a more advanced state of ECM remodelling compared with wild-type hearts and further substantiates our RNA-sequencing results indicating that SORBS2 affects ECM composition (*Figure 6K* and *L*). Despite the striking fibrotic phenotype due to *Sorbs2* loss, GSEA did not reveal terms that were directly related to fibroblast activation. Indeed, heatmap projection of the genes corresponding to GO term ‘fibroblast activation’ did not show a clear separation between wild-type and *Sorbs2*^−/−^ mice (*Figure 6M*). GSEA analysis did, however, indicate ‘epithelial–mesenchymal transition’ (EMT), an important contributor to cardiac fibrosis,^52^ to be enriched after the loss of *Sorbs2* under both baseline conditions and in response to TAB-induced stress (*Figure 6F*; Supplementary material online, *Figure S12A*). Heatmap projection of the expression of the core enrichment genes (this is the leading-edge subset of genes contributing the most to the enrichment result)^25^ of this term indicated the majority of these genes to be increased after loss of *Sorbs2*, which was further increased in combination with TAB (*Figure 6N*). This suggests that the fibrosis seen in the *Sorbs2*^−/−^ mice is more likely to be derived from EMT rather than fibroblast activation.

Integrins are well known to be associated with cardiac fibrosis.^8,47^ Our results show that the complete loss of *Sorbs2* in the adult heart leads to maladaptive changes that include ECM remodelling and integrin interactions. Given our demonstration of the interaction of SORBS2 with numerous proteins involved in the connection of the actin cytoskeleton to integrins, our data collectively indicate that *Sorbs2* is important for proper integrin function and as such is involved in the fibrotic response during heart failure.

**Figure 7:**
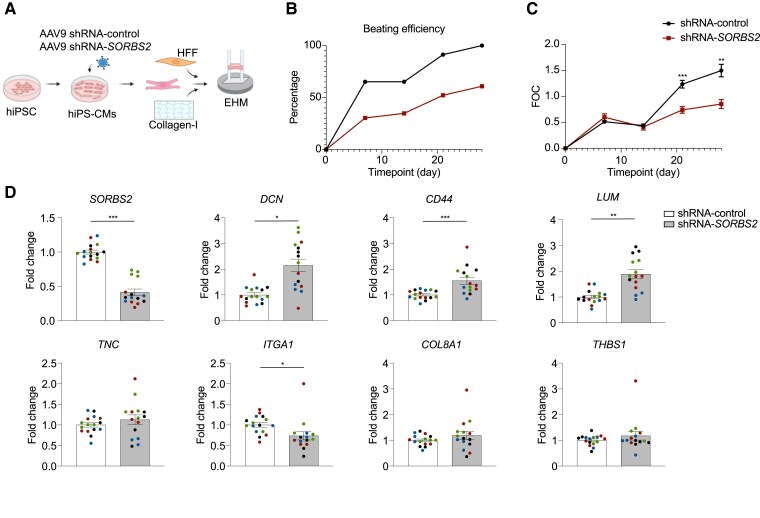
*SORBS2* knockdown in engineered human myocardium (EHM) impairs function. (*A*) Schematic overview of the experimental set-up. (*B*) Example of beating efficiency as indicated by percentage of beating EHMs over time. See Supplementary material online, *Figure S13* for remaining batches. (*C*) FOC of beating EHMs corresponding to *B*. (*D*) Gene expression changes measured by RT-qPCR. Each dot represents an EHM (*n* = 15–16) and are colour coded to experiment origin. Significance for FOC was measured by multiple two-tailed *t*-tests for each time point with Holm-Šídák adjustment for multiple comparisons. Significance for RT-qPCR was tested by a two-tailed nested *t*-tests where EHMs were grouped by experiment origin. Error bars: SEM. Significance levels: *P < 0.05, **P < 0.01, and ***P < 0.001. FOC, force of contraction; HFF, human foreskin fibroblast; hiPS-CMs, human-induced pluripotent stem cell- derived cardiomyocyte.

To study the effect of loss of *SORBS2* in human cardiomyocytes, we next employed an established model of engineered human myocardium (EHM)^53^ (*Figure 7A*). This 3D model consists of hiPS-CMs and human fibroblasts in a Type I collagen hydrogel. To diminish *SORBS2* expression, we used a combination of two different shRNAs targeting *SORBS2* and a non-targeting shRNA as control. Seven days before casting the EHMs, we transduced hiPS-CMs using adeno-associated virus to induce the expression of shRNAs. This led to a strong reduction in *SORBS2* resulting in a lowering of beating efficiency and a decrease in contraction force of the EHM (*Figure 7B* and *C*; Supplementary material online, *Figure S13*). Based on the localization pattern of SORBS2, our protein interaction data and RNA-sequencing data on hearts after the loss of SORBS2, we hypothesize that the diminished beating efficiency and force in our EHM could be a due to an effect of SORBS2 on the sarcomeres or cytoskeleton as hypothesized before^13,35^ or due to the effect of integrin interactions with the ECM on the contractile status of cardiomyocytes.^54^ The knockdown of *SORBS2* additionally corresponded to an increased expression of proteoglycan encoding genes such as Lumican (*LUM*) and Decorin (*DCN*) and the cell surface glycoprotein *CD44* (*Figure 7D*), which can all interact with Type I collagen,^55,56^ the main ECM component in this model. However, in this *in vitro* setting, we were unable to confirm several genes enriched in the *in vivo* situation such as Tenascin C (*TNC*), Integrin Alpha-1 (*ITGA1*), Collagen Type VIII Alpha 1 (*COL8A1*), and Thrombospondin 1 (*THBS1*) (*Figure 7D*). Taken together, using a 3D model of human myocardium, we found that reduced SORBS2 expression causes an inhibition in beating efficiency and force of contraction that corresponded to an increase in expression of ECM genes related to Type I collagen. These data further support a functional role for SORBS2 in ECM composition that is conserved across species.

## 4. Discussion

In this study, we show the power of combining independent single-cell sequencing data sets focusing on heart failure in both mice and humans, to identify genes associated with cardiomyocyte stress across species. Using this approach, we not only recognized well-established marker genes of cardiomyocyte failure but also less well-studied genes linked to this condition, including *Sorbs2*. Follow-up analysis indicated the robust elevated expression of *Sorbs2* upon stress, which correlated to phenotypical hallmarks of heart failure both in mice and human patients. Additionally, we identified the primary promotor region for the stress-induced elevation of *Sorbs2* expression within the *Sorbs2* mouse and human locus and demonstrated that GATA4 can regulate *Sorbs2* expression at this region. Furthermore, by uncovering the interaction partners of SORBS2 and studying the effect of SORBS2 loss in healthy and diseased cardiomyocytes, we show that SORBS2 is a crucial regulator of integrin interactions and fibrosis.

SORBS2 has previously been linked to cardiac function and disease. While originally it was proposed that SORBS2 expression was specifically induced during left ventricular non-compaction cardiomyopathy,^14^ we and others showed its induction to be more common and shared in a wider variety of cardiomyopathies.^13^ Compromised *Sorbs2* expression has been shown to contribute to cardiac malformations in patients with 4q deletion syndrome.^35^ Global loss of SORBS2 in mice resulted in a phenotype resembling ARVC with accompanying fibrosis,^12^ while cardiomyocyte-specific deletion resulted in a slow-progressing DCM phenotype.^13^ Because of the robust induction of *Sorbs2* expression specifically upon stress and its association with heart failure, we here utilized an inducible knockout model to study the role of *Sorbs2* in the context of heart failure. In our inducible model, the loss of *Sorbs2* induced a slight increase in cardiomyocyte hypertrophy and a non-significant trend in fibrosis. However, under cardiac stress conditions, the fibrotic response became excessive in the absence of SORBS2 while mice also displayed a severe decrease in left ventricular volume. These phenotypical effects observed in response to SORBS2 loss in the different mouse models indicate SORBS2 to function as a fundamental protein in the cardiac remodelling process during heart disease. In addition, *Sorbs2*^−/−^ mice also displayed left atrial enlargement under TAB-induced cardiac stress (*Figure 5E* and *F*). This is in line with a previous report showing that the cardiomyocyte-specific deletion of *Sorbs2* results in left atrial enlargement, with considerable increases in atrial size observed from about 6 months of age. Because analyses in this study were conducted using ventricular tissue, future research is required to study the chamber-specific effects of SORBS2. The mouse model with constitutive cardiomyocyte-specific deletion of *Sorbs2* has also been exposed to TAB.^13^ Although fibrosis was not assessed here, TAB did not induce an additional functional defect in the *Sorbs2* knockout mouse, as measured by EF and cardiac mass. As our inducible cardiomyocyte-specific deletion model did show exaggerated pathological remodelling in response to TAB, we hypothesize that there might be a compensatory mechanism activated in the mice where SORBS2 is deleted in a constitutive manner. Additionally, given the sex-related differences in cardiac ECM composition,^57^ it might be of particular interest to examine sex-specific roles of SORBS2 in the future.

Our data indicate a role for SORBS2 in integrin interactions and ECM composition. Previously, it has been suggested that SORBS2 is involved in microtubule polymerization.^13,14^ Given the close relationship of actin dynamics, integrins, and microtubule dynamics,^58–60^ it is likely that the effects seen on microtubule polymerization are a result of this complex interplay. A recent study from McLendon *et al*.^13^ showed that mice with continuous cardiomyocyte-specific loss of *Sorbs2* exhibited an age-dependent increase of integrin beta-1 expression, becoming apparent after 1 year. While we did not find an increase in *Itgb1* in our *Sorbs2*^−/−^ compared with wild-type mice under baseline conditions, our RNA-sequencing data did indicate an enrichment of integrins that can form heterodimers with ITGB1. These data imply that *Sorbs2* loss affects a range of integrins relatively early while the specific integrins affected can be time and situation dependent. Furthermore, we demonstrate that the genetic loss of *Sorbs2* induces changes in ECM composition, which are exacerbated during cardiac disease. We found that *Sorbs2* loss leads to an increase in more matured cross-linked collagen fibres, which is associated with myocardial stiffness,^61^ suggestive of a regulatory role for *Sorbs2* on myocardial stiffness.

GATA4 is an important transcriptional regulator of many genes enriched in cardiomyocytes, involved in actin cytoskeletal organization,^42^ and its transcriptional activity is elevated in response to mechanical stress.^39,40^ Our observations that *Sorbs2* is highly enriched in cardiomyocytes, binds to proteins involved in actin cytoskeletal organization, and is increased in response to stress make GATA4 a feasible candidate to be involved in its transcriptional activation. Moreover, we showed that treatment of hiPS-CMs with ET-1, a known GATA4 activator,^43^ increased the expression of SORBS2 while GATA4 knockdown had an opposing effect. Also, as *Nppa* is a transcriptional target of GATA4,^41^ and we found *Sorbs2* and *Nppa* expression to be correlated, our results strongly suggest that GATA4 activation is at least partially responsible for the increased expression of *Sorbs2* and *Nppa* in stressed cardiomyocytes. However, at this point, we cannot exclude the contribution of other potential (co-)factors or genomic regions involved in the regulation of *Sorbs2*.

Our interaction partner data confirmed some known cardiac-binding proteins of SORBS2^62^ and provided extended insights on more unknown cardiac interaction partners. Perhaps the most surprising interaction partners of SORBS2 are the proteins that make up the WRC. The WRC consists of five proteins (CYFIP1 or CYFIP2; NCKAP1 or NCKAP1l; WASF1, WASF2, or WASF3; ABI1, ABI2, or ABI3; and BRCK1) and is a key regulator of actin polymerization by regulating ARP2/3.^46^ As actin dynamics are highly studied in the field of migrating cells where the cytoskeleton is continuously remodelled, many of the knowledge regarding the WRC is derived from studies in this setting. To the best of our knowledge, this protein complex is not yet well characterized in adult cardiomyocytes. Consistent with our findings in the *Sorbs2*^−/−^ mice, the binding proteins of SORBS2 support its role as adaptor protein in connecting integrins to the actin cytoskeleton. Additionally, the WRC has been demonstrated as important facilitator of this connection.^45^ Our data suggest that SORBS2, the WRC, and other binding proteins affect integrin interactions and as such underlies a fibrotic response in the heart. This is in line with previous studies showing that integrins in the heart play a critical role in regulating fibrosis.^8,47^

Adaptor proteins involved in integrin–cytoskeletal connections (indirectly) impact on the cytoplasmic tail of integrins. This leads to conformational changes in the extracellular domain of integrins, altering their affinity for ECM ligands, a process that is known as inside-out signalling. Although future research is required to delineate the complete cascade through which SORBS2 affects fibrosis, several studies support our notion that this is mediated via changed integrin interactions. Cardiomyocyte loss of KINDLIN-2,^63^ TALIN-1 and TALIN-2,^64^ or VINCULIN,^65^ all cytoskeletal–integrin adaptor proteins, induces fibrosis. These proteins are all known to mediate integrin activation, and their depletion from cardiomyocytes was accompanied by alterations in integrin expression. Also, similar to our findings, genetic loss of *Zyxin*, one of the identified SORBS2-binding proteins and another integrin–cytoskeletal adaptor protein, likewise induces a slight fibrotic phenotype that is exacerbated after pathological remodelling.^66^ We hypothesize that a changed expression in SORBS2 disturbs the homeostasis of these multi-protein complexes and subsequent integrin activation. Together, this work emphasizes the importance of integrin–cytoskeletal adaptor proteins in the heart and their role in fibrotic remodelling.

The positive association between *Sorbs2* expression and fibrosis in heart failure, and the exacerbated fibrotic response after homozygous loss of *Sorbs2*, suggests that imbalanced levels of SORBS2 are detrimental in the context of cardiac stress. Indeed, the cardiac overexpression of *Sorbs2* has also been shown to be detrimental for cardiac function.^14^ A similar phenomenon has been described for *Col5a1*, of which the expression increases after cardiac injury and correlates to cardiac function, whereas the homozygous loss of *Col5a1* exacerbates the fibrotic response after injury in an integrin-dependent manner.^8^ Together with our data, this underlines the importance and complexity of genes associated with integrin interactions in cardiac disease.

In conclusion, our study shows that SORBS2 is fundamentally associated with cardiac remodelling as an adaptor protein that regulates integrin interactions and cardiac fibrosis. These findings emphasize the importance of adaptor proteins and integrins in pathological cardiac remodelling, which aids the understanding of molecular processes underlying heart failure and provides insight for future therapeutics.

**Translational perspective**

*Sorbs2* expression is robustly increased in heart failure and correlates to the severity of the disease, while complete loss of *Sorbs2* is also pathological, emphasizing the importance of tightly controlled levels of this gene to prevent cardiac pathology. SORBS2 is an adaptor protein that regulates integrin interactions and cardiac fibrosis. This study highlights the importance of adaptor proteins and integrins during pathological cardiac remodelling, which enhances the comprehension of molecular processes driving heart failure and provides insight for future therapeutics.

## Supplementary Material

cvaf021_Supplementary_Data

## Data Availability

The authors declare that the main data supporting the findings of this study are available within the article and its Supplementary material. Generated RNA-sequencing raw and processed data are deposited in the Gene Expression Omnibus (GEO) repository and available under accession code: GSE243463.
