# Supplementary material for: Cardiomyocyte SORBS2 expression increases in heart failure and regulates integrin interactions and extracellular matrix composition
Source: Cardiovasc Res. 2025 Feb 17;121(4):585–600. doi: 10.1093/cvr/cvaf021 (PMC12054630; doi:10.1093/cvr/cvaf021)
Supplement: cvaf021_Supplementary_Data [file cvaf021_supplementary_data.pdf]

Supplementary materials

Cardiomyocyte SORBS2 expression increases in heart failure and regulates integrin interactions and ECM composition.

Timmer et al.

Content

Supplementary figure 1-13
Supplementary table 1-9
Supplementary methods

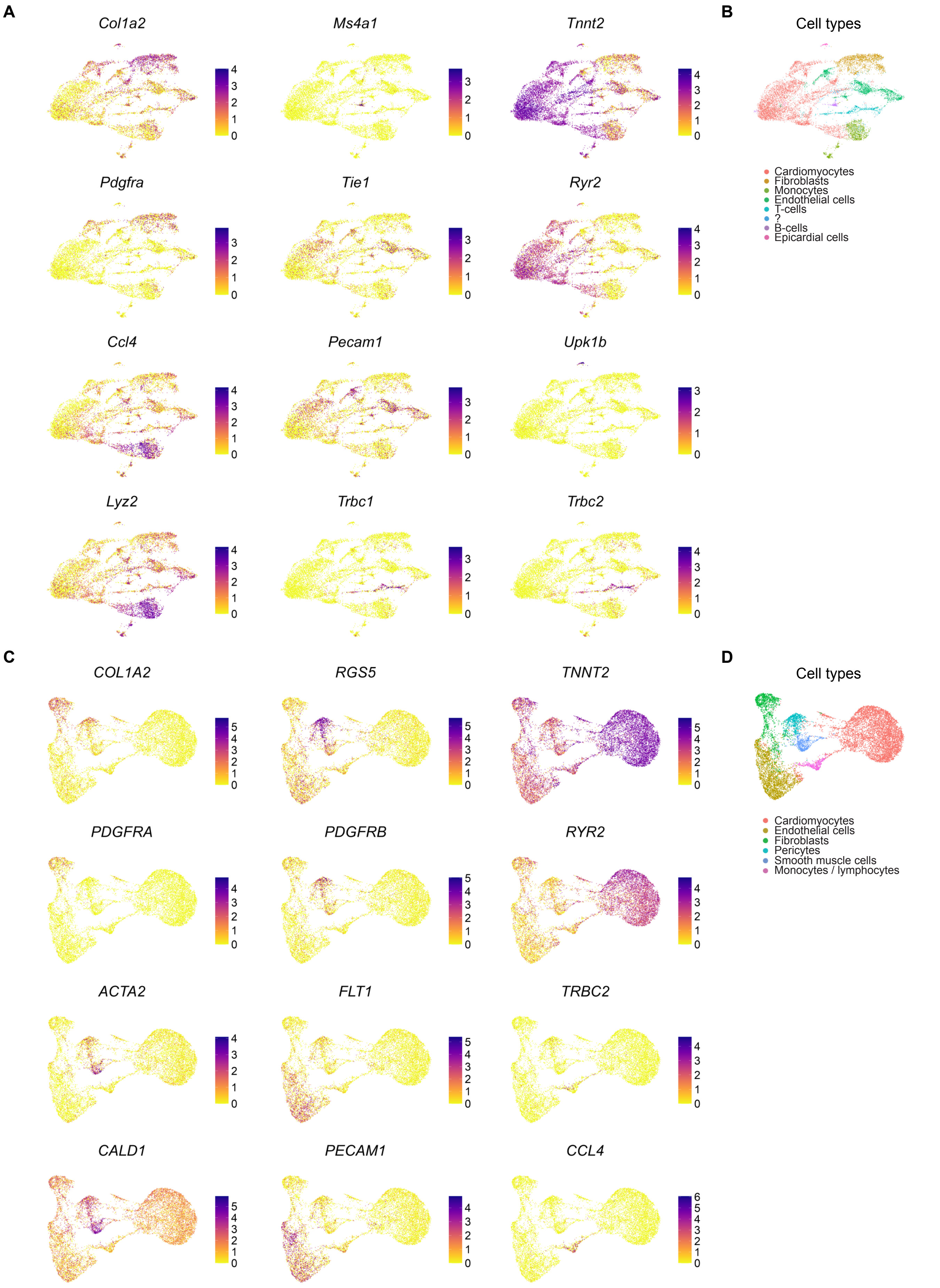

Fig. S1. Single-cell transcriptomic cell type marker genes (A) Uniform Manifold Approximation and Projection (UMAP) plots for all cells (n = 11492) present in murine dataset corresponding to Fig. 1, in which cells are color coded by expression for different cell type markers. **(B)** UMAP plot indicating different cell types in murine dataset. Same as left panel Fig. 1B, also shown here for clarity. **(C)** UMAP plots for all cells (n = 12861) present in human dataset corresponding to Fig. 1, in which cells are color coded by expression for different cell type markers. **(D)** UMAP plot indicating different cell types in human dataset. Same as left panel Fig. 1C, also shown here for clarity.

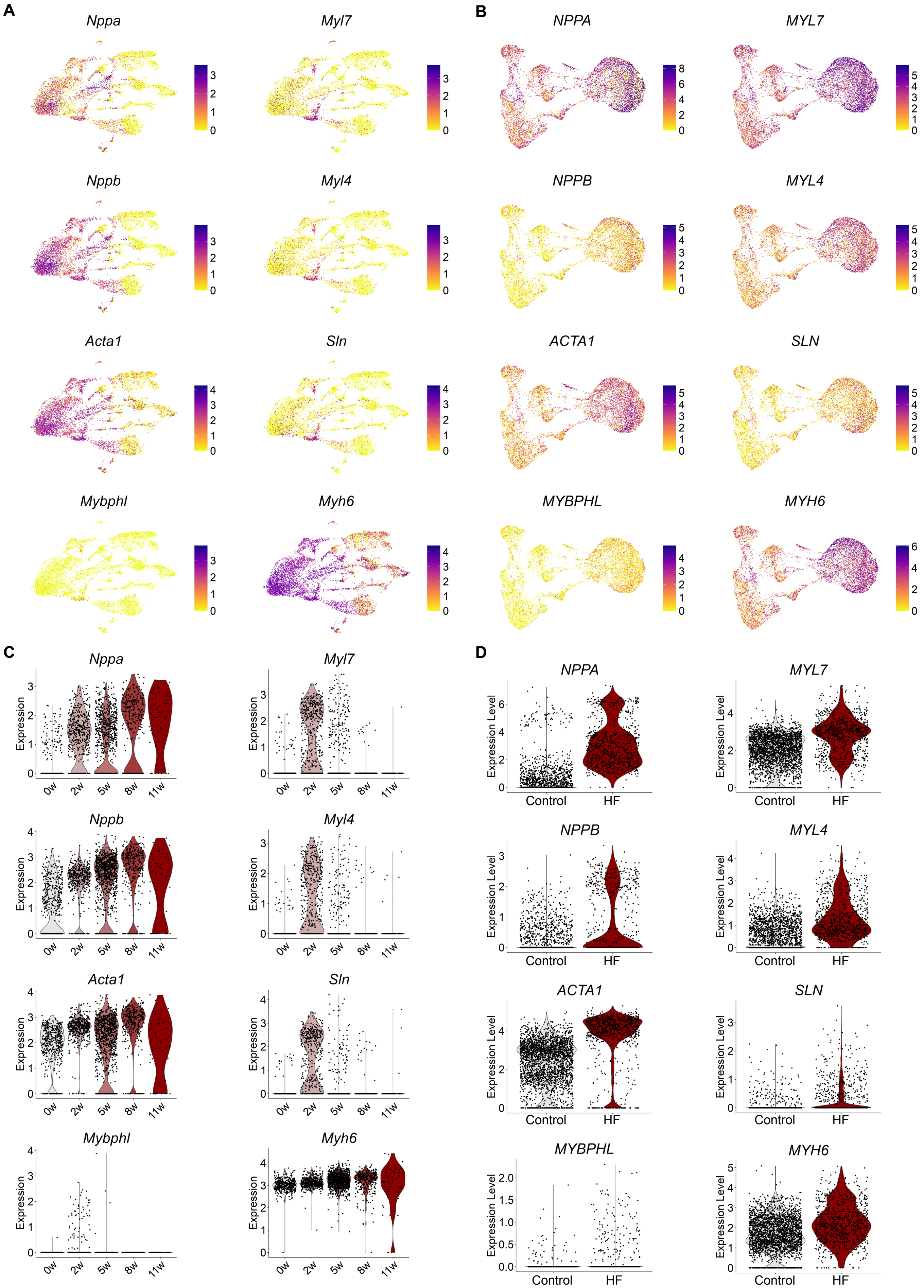

Fig. S2. Single cell RNA expression pattern of top ranked NPPA correlated genes. (A) Uniform Manifold Approximation and Projection (UMAP) plots for all cells (n = 11492) present in murine dataset corresponding to Fig. 1, in which cells are color coded by expression for the top ranked conserved *Nppa* correlated genes indicated in the venn diagram of Fig. 1, excluding *Sorbs2*. **(B)** UMAP plots for all cells (n = 12861) present in human dataset corresponding to Fig. 1, in which cells are color coded by expression for the top ranked conserved *NPPA* correlated genes indicated in the venn diagram of Fig. 1, excluding *SORBS2*. **(C)** Violin plots for the expression of the same genes as in A in mouse cardiomyocytes (n = 2298), in which different timepoints indicate weeks post transverse aortic banding (TAB). **(D)** Violin plots for the expression of the same genes as in A-C in human cardiomyocytes (n = 3253) originating from failing (HF) or control hearts.

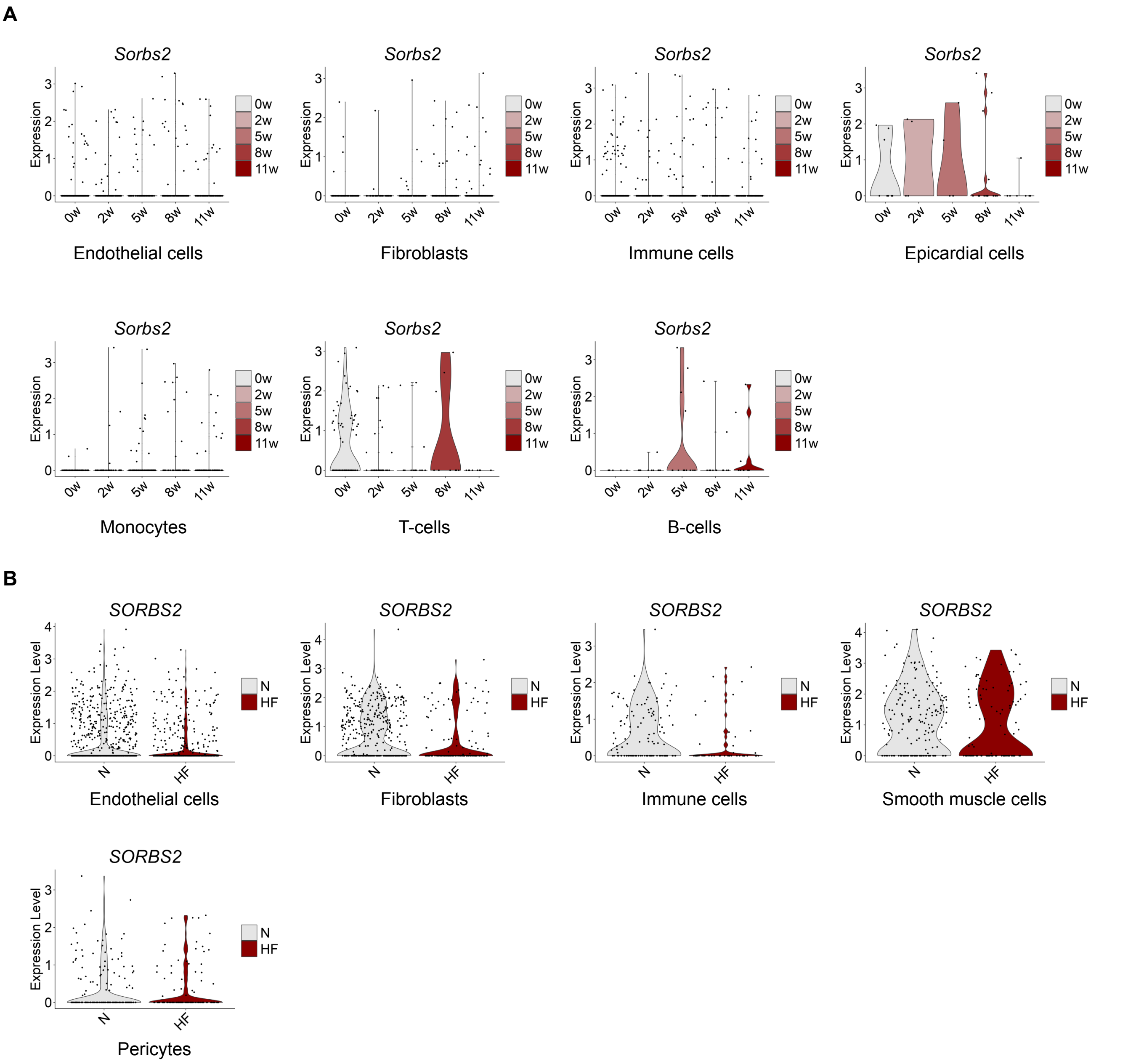

Fig. S3. ***SORBS2*** **expression per cell type in healthy and diseased hearts (A)** Violin plot of *Sorbs2* expression in different cell types at different timepoints (weeks) after transverse aortic banding (TAB). Endothelial cells: n = 671. Fibroblasts: n = 523. Immune cells: n = 639. Epicardial cells: n = 40. Monocytes: n = 428. T-cells: n = 155. B-cells: n = 56. **(B)** Violin plot of *SORBS2* expression in different cell types from control or failing (HF) hearts. Endothelial cells: n = 1274. Fibroblasts: n = 549. Immune cells: n = 177. Smooth muscle cells: n = 291. Perictyes: n = 300.

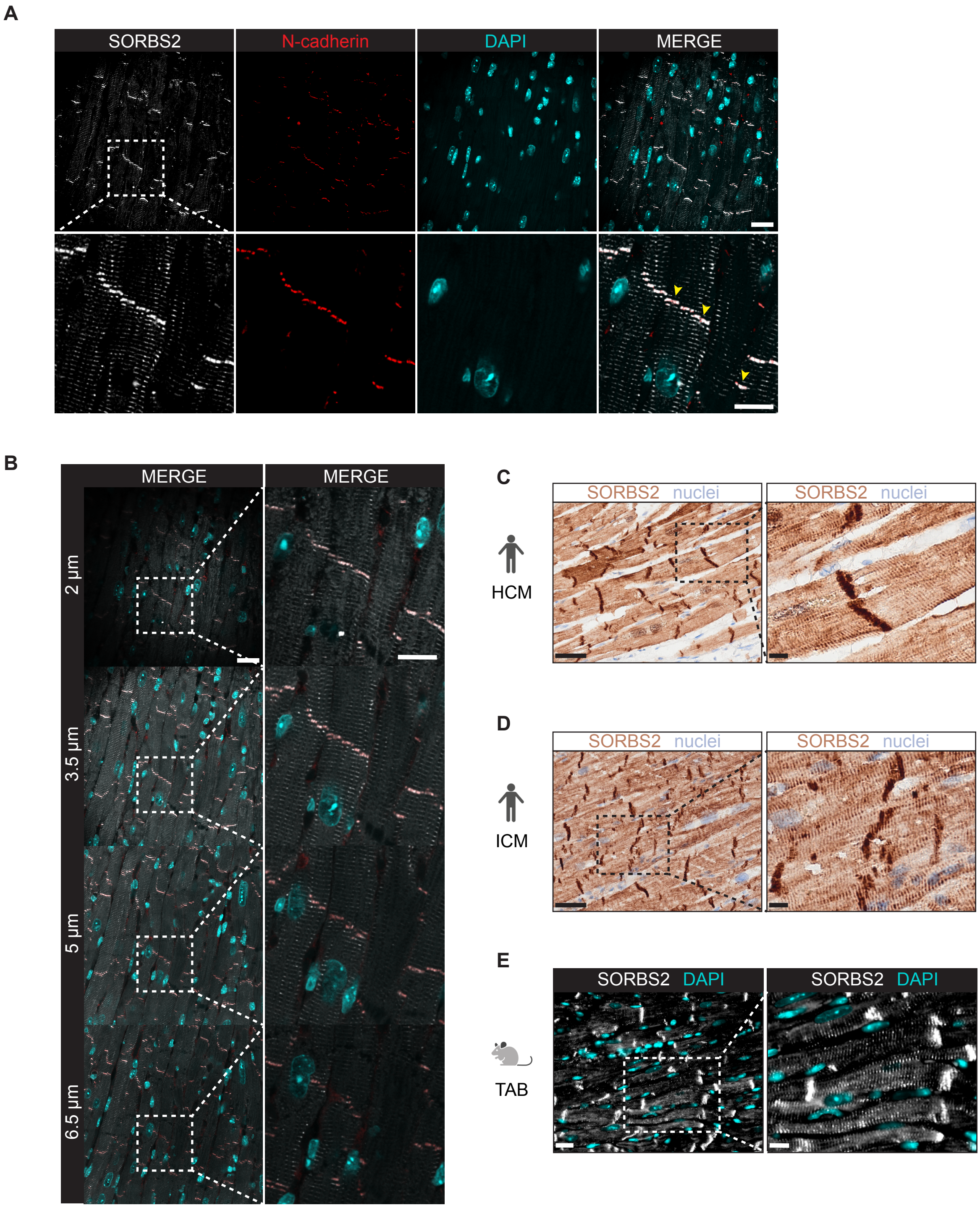

Fig. S4. SORBS2 localizes at intercalated disc and does not change localization during disease. (A) SORBS2 colocalization with the intercalated disc marker N-cadherin in mouse cardiac tissue. Scalebar upper panels: 20µm. Scalebar lower panels: 10µm. Arrowheads indicate example spots of colocalization. **(B)** Seperate z-stack images of example shown in A. Distance in z-stack indicated on the left. **(C-D)** SORBS2 immunostaining on diseased human cardiac tissue derived from hypertrophic cardiomyopathy (HCM) (B) and ischemic cardiomyopathy (ICM) (C). Scalebar left panel 50µm. Scalebar right panel: 10mm. **(E)** SORBS2 immunostaining on diseased mouse cardiac tissue induced by transverse aortic banding (TAB). Timepoint of tissue collection: 11 weeks post-TAB. Scalebar left panel 20µm. Scalebar right panel: 10µm.

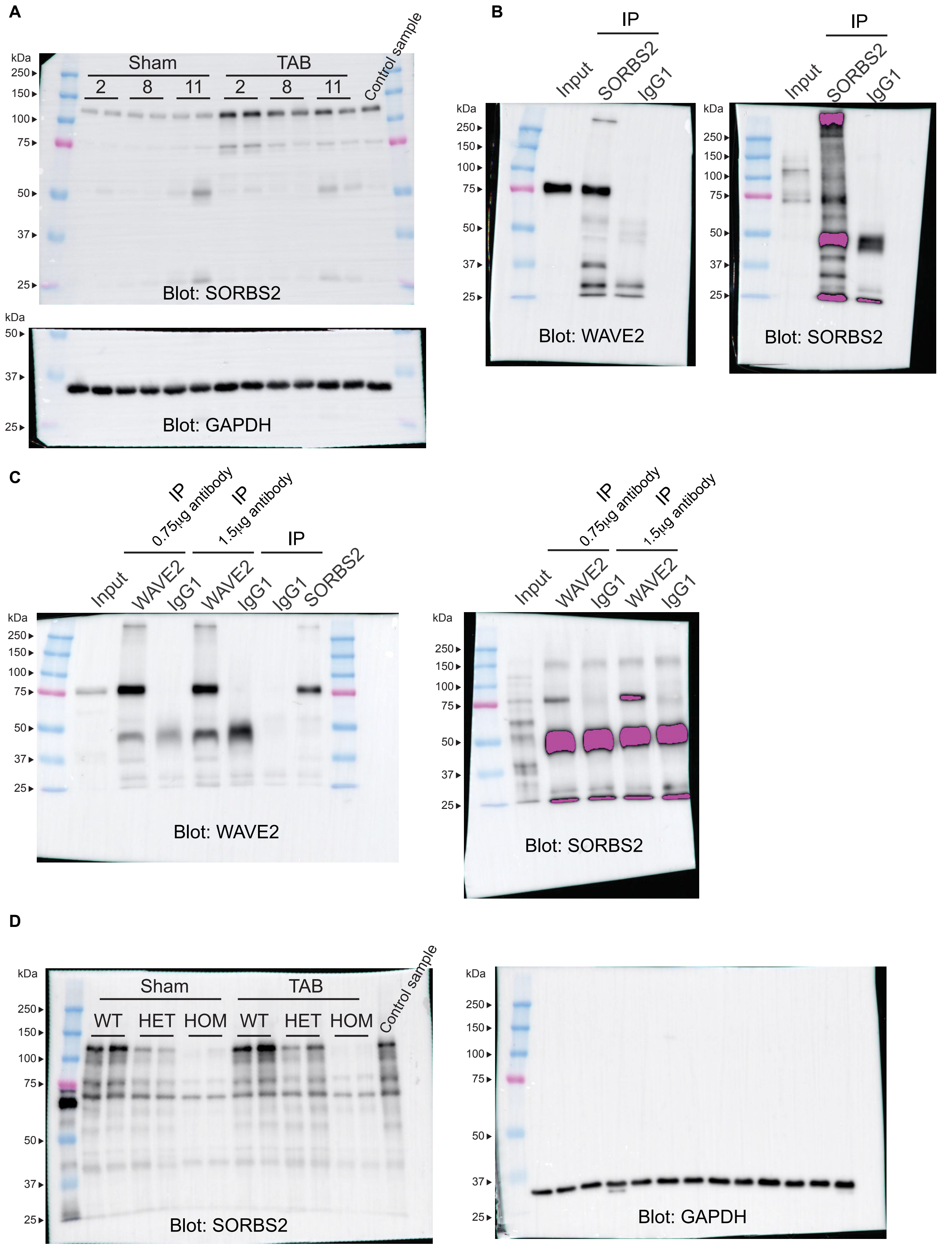

Fig. S5. Full images of Western Blots shown in main figures (A) Full images corresponding to Fig. 2C **(B)** Full images corresponding to Fig. 4E **(C)** Full images corresponding to Fig. 4F **(D)** Full images corresponding to Fig. 5C.

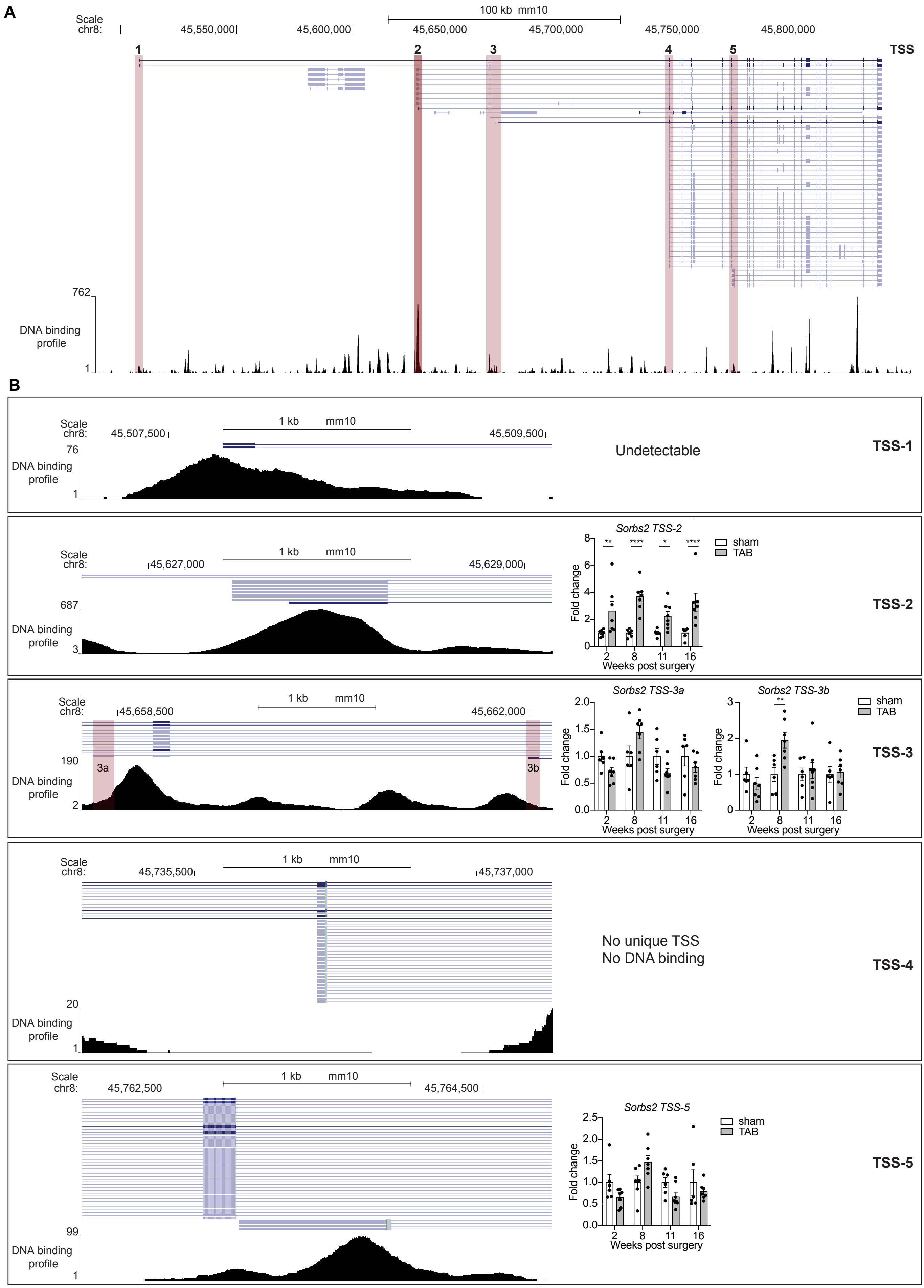

Fig. S6. TAB induces SORBS2 expression from transcription start site (TSS) 2. (A) Schematic overview of the Sorbs2 locus in the mouse reference genome and density plot of DNA binding at this region derived from the ReMap atlas. Genomic regions around a transcription start site (TSS) are marked in red, TSS-2 is marked in dark red. Enlarged view of Fig. 3A, also shown here for clarity. **(B)** Zoom-in images of different TSS regions marked red in A (left) and mRNA expression of transcripts from these TSS (right) by RT-qPCR at different timepoints after transerse aortic banding (TAB). TSS-1: undetectable signal with 2 different primer pairs. TSS-2: This site is used as mRNA expression in Fig. 2B, also shown here for clarity. TSS-3: two different TSS in this region are present and indicated as TSS-3a and TSS-3b. TSS-4: due to the genomic structure it is not possible to design primers unique to this TSS. However, there is also no DNA binding at this TSS region detected. Dots represent biological replicates (n = 6-8 per condition). Two-way ANOVA with Šidák's multiple comparisons test was used for statistical testing to corresponding sham timepoint, *p<0.05, **p<0.01, ****p<0.0001.

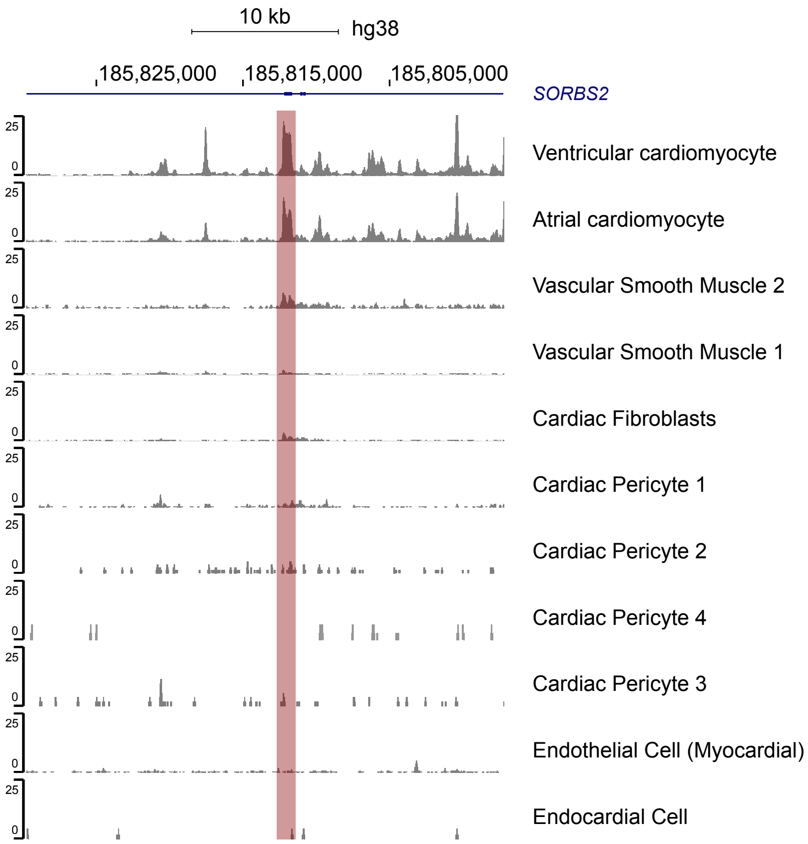

Fig. S7. Single-cell chromatin accessibility of SORBS2 TSS-2. Human single-cell chromatin accessibility of the genomic region surrounding TSS-2 corresponding to Fig. 3D from cardiac cell types present in the cis-element ATLAS. Genomic region around TSS-2 is marked in red.

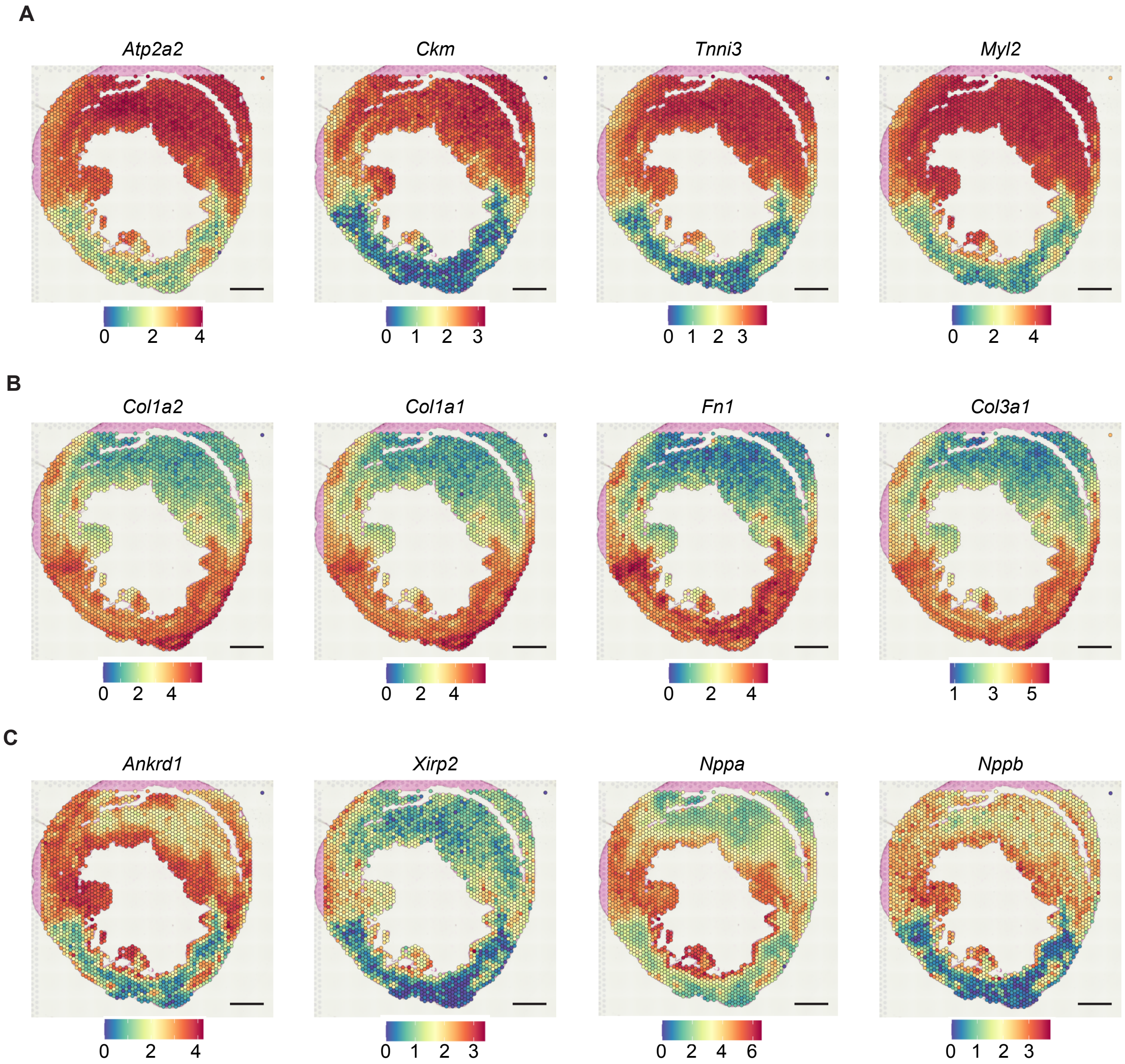

Fig. S8. Spatial transcriptomic analysis to identify different zones after myocardial infarction (MI). (A) Spatial expression of remote zone marker genes underlying Fig. 3H. Scalebar: 1mm. **(B)** Spatial expression of infarct zone marker genes underlying Fig. 3H. Scalebar: 1mm. **(C)** Spatial expression of border zone marker genes underlying Fig. 3H. Scalebar: 1mm.

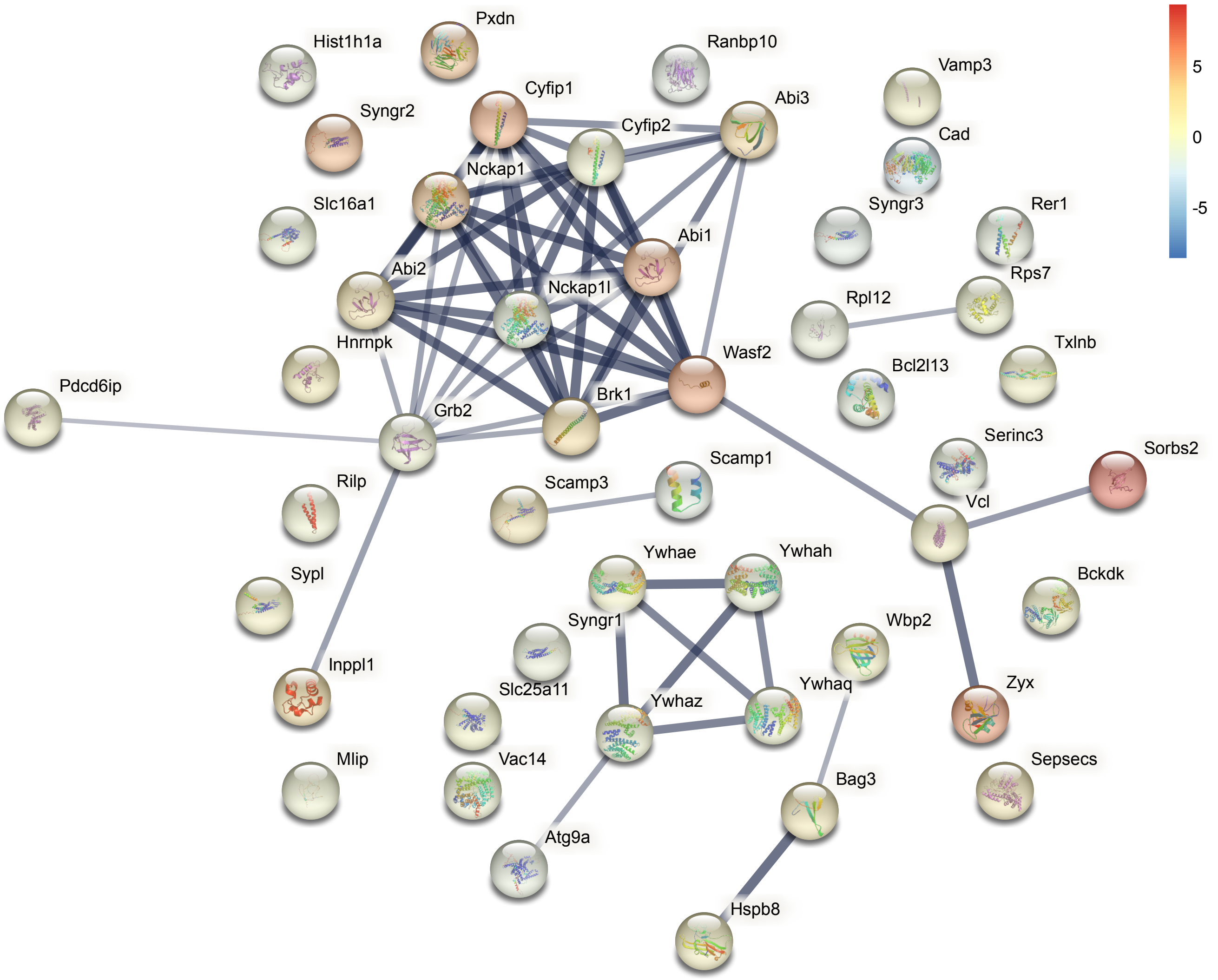

Fig. S9. Complete SORBS2 interactome. STRING interaction map of all significant cardiac SORBS2 binding proteins. Edges between nodes are based on the STRING's confidence setting and indicate strength of data support for the connected nodes, with thicker edges indicating stronger data support. Nodes are colored by entering color code of the abundance of each protein within the SORBS2 IP corresponding to the heatmap color scale in Fig. 4. Note, shading and opacity of the nodes induces shade differences from the scalebar in Fig. 4.

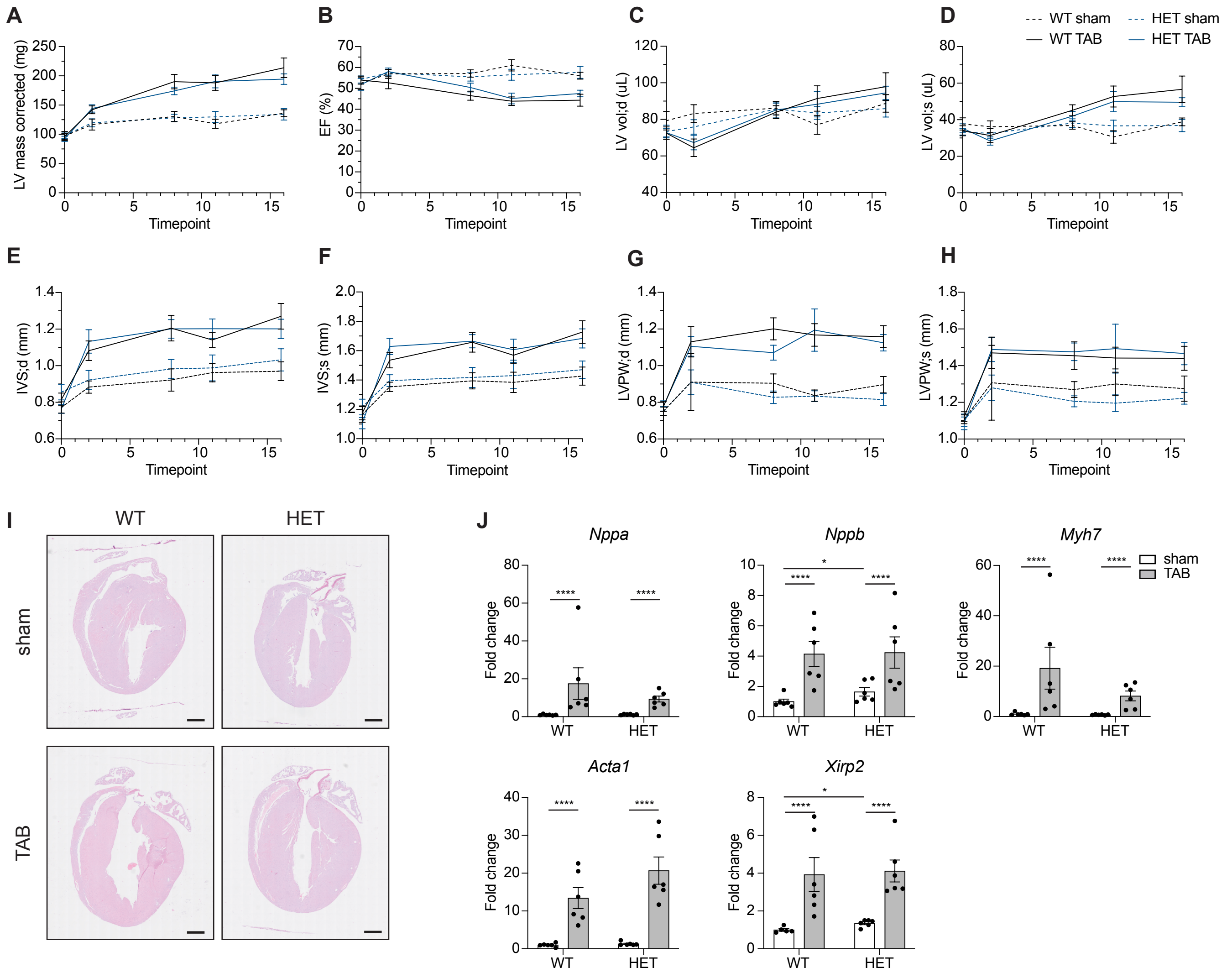

Fig. S10. Heterozygous loss of Sorbs2 does not affect cardiac function post-TAB. (A-H) Echocardiographical output corresponding to experimental set-up show in Fig. 5A for wildtype (WT) and Sorbs2$^{+/-}$ (HET) mice, n = 8-14 per condition per timepoint. TAB: transverse aortic banding. LV mass corrected: corrected LV mass derived from echocardiography analysis, EF: ejection fraction, LV vol: left ventricular volume at end of diastole (;d) or end of systole (;s), IVS: inter ventricular septal thickness at diastole (;d) or systole (;s), LVPW, left ventricular posterior wall thickness at diastole (;d) or systole (;s). **(I)** Cardiac morphology showed by representative examples of hematoxylin and eosin staining. WT images same as Fig. 5F, also shown here for clarity. Scalebar: 1mm. **(J)** mRNA expression of cardiac stress related genes by RT-qPCR. Dots indicate biological replicates (n = 6). Significance was tested by an ordinary two-way ANOVA with Tukey's multiple comparisons test. Note, for Nppa and Myh7 not all required assumptions were met. For clarity, significance of primary comparisons of interest are shown. Error bars: standard error of the mean. Significance levels: *p<0.05, ****p<0.0001.

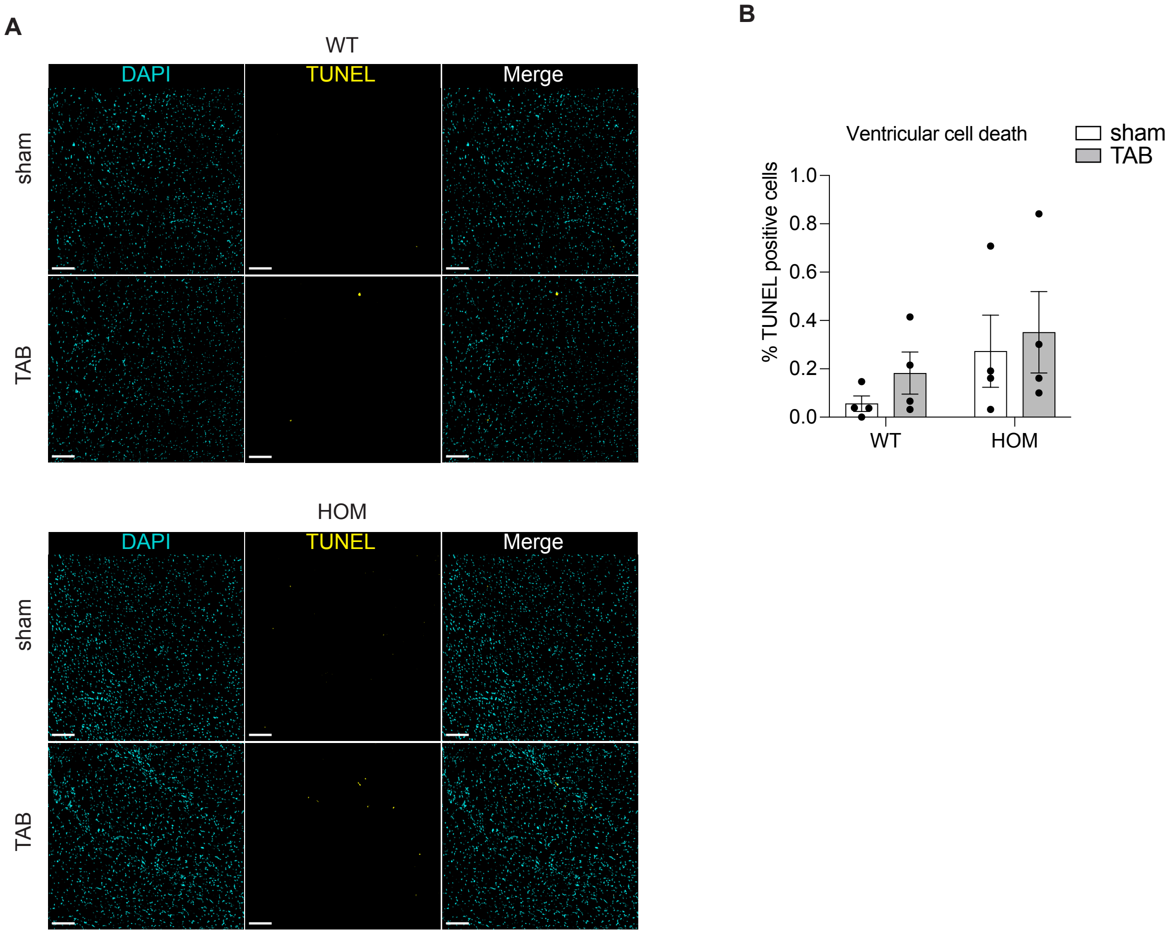

Fig. S11. TUNEL assay in mice hearts. (A) Representative images of the left ventricle of wildtype (WT) mice or Sorbs2-/- (HOM) mice after TUNEL assay. TAB: transverse aortic banding. WT: wildtype. Scalebar: 100µm (B) Quantification of A, dots represent biological replicates (n = 4). No significant differences as determined by a two-way ANOVA. Error bars: standard error of the mean.

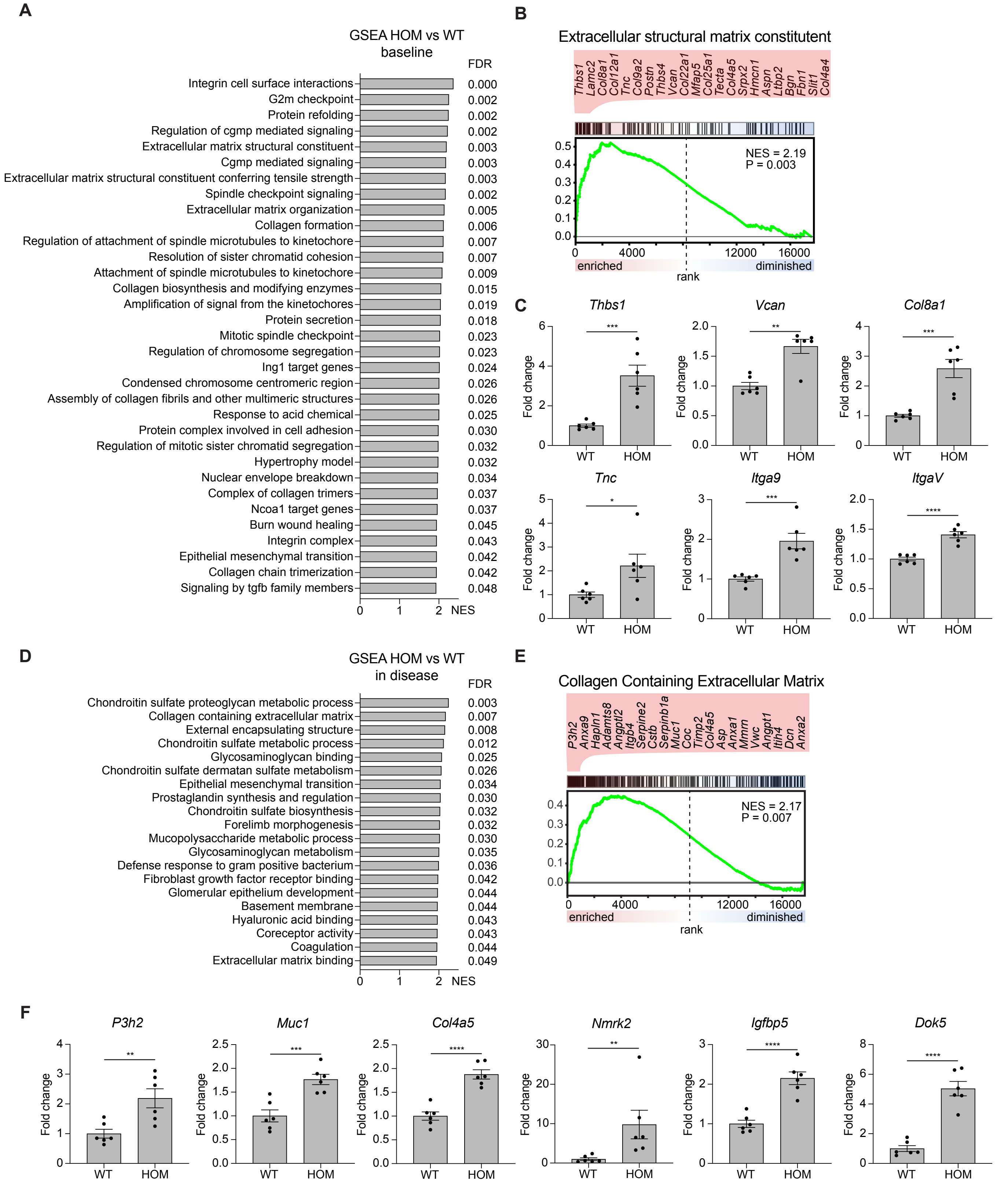

Fig. S12. SORBS2 loss induces an extracellular matrix gene program. (A) All significant Gene Set Enrichment Analysis (GSEA) terms ordered by normalized enrichment score (NES) resulting from comparison of Sorbs2$^{-/-}$ (HOM) and wildtype (WT) mice in healthy setting. FDR: False discovery rate. **(B)** Enrichment plot of the first extracellular matrix containing term in A. **(C)** Validation by RT-qPCR of several genes indicated as enriched in HOM compared to WT mice under baseline conditions by RNA-seq. Dots represent biological replicates (n = 6). **(D)** All significant GSEA terms orderd by NES resulting from comparison of HOM and WT mice 16 weeks after transverse aortic banding (TAB). FDR: False discovery rate. **(E)** Enrichment plot of the first extracellular matrix containing term in D. **(F)** Validation by RT-qPCR of several genes indicated as enriched in HOM compared to WT mice 16 weeks after TAB by RNA-seq. Dots represent biological replicates (n = 6). Statistical tests used in C and F: unpaired student's t-test (two-sided) or Mann-Whitney U test (two-sided). Error bars: standard error of the mean. Significance levels: *p<0.05, **p<0.01, ***p<0.001, ****p<0.0001.

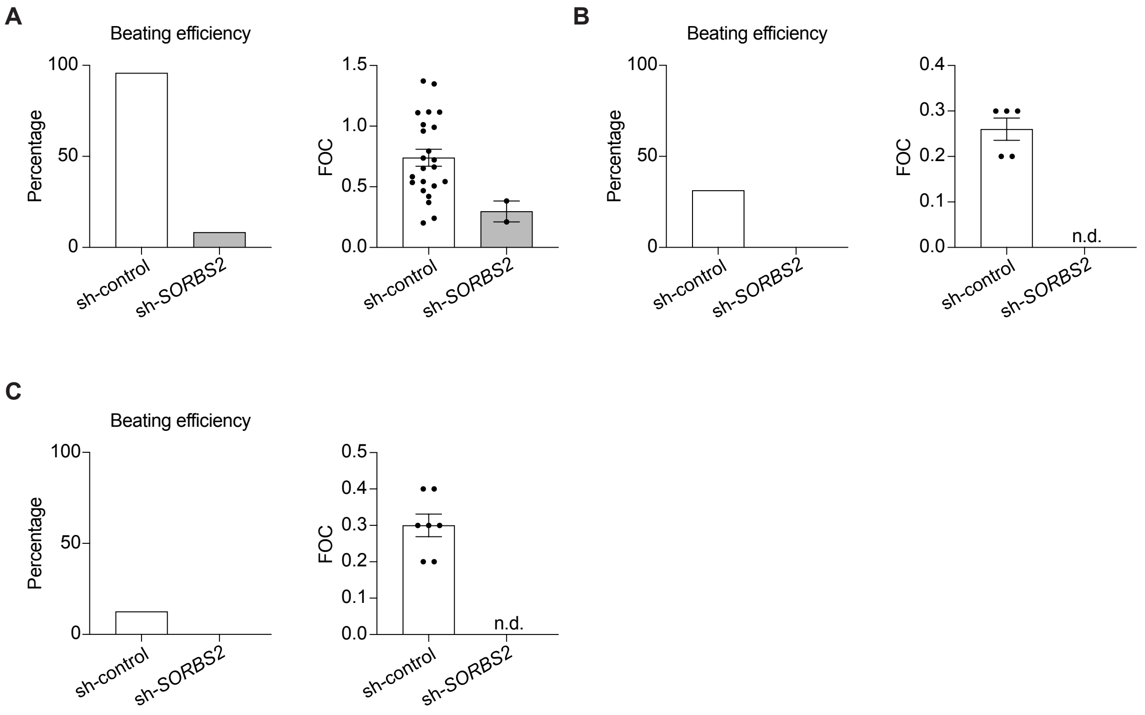

Fig. S13. Sorbs2 knock-down in human engineered heart muscle (EHM) impairs function. (A-C) Percentage of beating EHMs measured at day 28 from an independent differentiation and casting (left) and corresponding force of contraction (FOC) of beating EHMs (right). n.d.: not determined. Error bar: standard error of the mean.

TIMEPOINT	2 WEEKS		8 WEEKS		11 WEEKS		16 WEEKS	
	sham (n=9)	TAB (n=11)	sham (n=9)	TAB (n=11)	sham (n=9)	TAB (n=12)	sham (n=9)	TAB (n=11)
IVS;d (mm)	0.81 ± 0.08	1.03 ± 0.09*	0.85 ± 0.10	1.10 ± 0.20*	0.82 ± 0.10	1.07 ± 0.16*	0.78 ± 0.09	1.15 ± 0.17*
IVS;s (mm)	1.13 ± 0.10	1.38 ± 0.11*	1.15 ± 0.15	1.45 ± 0.21*	1.19 ± 0.15	1.37 ± 0.15*	1.23 ± 0.14	1.44 ± 0.18*
LVID;d (mm)	4.10 ± 0.18	3.95± 0.33	4.38 ± 0.29	4.73 ± 0.54	4.21 ± 0.31	4.49 ± 0.30	4.37 ± 0.21	4.93 ± 0.69
LVID;s (mm)	2.97 ± 0.21	2.86 ± 0.38	3.26 ± 0.27	3.78 ± 0.77	2.96 ± 0.34	3.70 ± 0.32*	3.04 ± 0.20	4.14 ± 0.86*
LVPW;d (mm)	0.79 ± 0.21	0.97 ± 0.17	0.77 ± 0.18	0.96 ± 0.29	0.69 ± 0.14	0.98 ± 0.17*	0.69 ± 0.11	0.88 ± 0.16*
LVPW;s (mm)	1.07 ± 0.20	1.29 ± 0.32	0.97 ± 0.21	1.17 ± 0.29	0.95 ± 0.17	1.03 ± 0.17	0.97 ± 0.12	1.02 ± 0.14
EF (%)	53.92 ± 6.32	54.32 ± 8.22	50.55 ± 5.28	41.34 ± 14.48	57.17 ± 5.52	36.41 ± 6.92*	57.80 ± 5.68	34.48 ± 12.37*
FS (%)	27.66 ± 4.15	27.9 ± 5.18	25.61 ± 3.27	20.67 ± 8.30	29.81 ± 3.60	17.48 ± 3.79*	30.34 ± 3.75	16.73 ± 6.60*
LV mass (mg)	122.13 ± 12.09	158.30 ± 28.44*	141 ± 43.74	224.93 ± 82.03*	119.81 ± 23.94	199 ± 36.78*	121.82 ± 13.01	236.78 ± 78.44*
LV mass corrected (mg)	97.71 ± 9.67	126.64 ± 22.75*	113.21 ± 34.99	179.94 ± 65.62*	95.85 ± 19.11	159.71 ± 29.43*	97.45 ± 10.41	189.42 ± 62.76*
LV vol;d (µl)	74.60 ± 7.67	68.66 ± 13.45	87.08 ± 13.39	105.54 ± 28.11	79.52 ± 14.26	92.33 ± 13.82	86.45 ± 9.98	117.28 ± 36.88
LV vol;s (µl)	34.39 ± 5.81	31.92 ± 10.26	43.17 ± 8.61	64.81 ± 30.14	34.54 ± 9.82	58.90 ± 11.35*	36.43 ± 6.00	80.50 ± 36.96*
HW/BW	4.79 ± 0.15	6.51 ± 0.78*	4.95 ± 0.36	8.27 ± 1.85*	4.99 ± 0.39	7.20 ± 0.78*	4.41 ± 0.37	7.97 ± 1.54*
HW/TL	7.41 ± 0.35	10.44 ± 1.43*	8.81 ± 0.65	14.07 ± 3.51*	9.05 ± 0.79	13.06 ± 1.82*	8.76 ± 0.82	14.81 ± 3.25*

Table S1. Functional measurements at different timepoint after transverse aortic banding (TAB). IVS: inter ventricular septal thickness at diastole (;d) or systole (;s), LVID: left ventricular internal dimension at diastole (;d) or systole (;s), LVPW, left ventricular posterior wall thickness at diastole (;d) or systole (;s), EF: ejection fraction, FS: fractional shortening, LV mass: left ventricular mass derived from echocardiography analysis, LV mass corrected: Corrected LV mass derived from echocardiography analysis, LV vol;d: left ventricular end diastolic volume, LV vols: left ventricular end systolic volume, HW/BW: heart weight to body weight ratio, HW/TL: heart weight to tibia length ratio. *Significant ($p<0.05$) different from corresponding sham. ± indicates standard deviation. Statistical analysis for FS, LVPW;d, LVPW;s, IVS;s: Two-way ANOVA with Šidák's multiple comparisons test. Statistical analysis for EF, HW/BW, HW/TL, LVEDV, LVESV, LVID;d, LVID;s, LV mass, LV mass corrected, IVS;d: multiple Mann-whitney tests with Holm- Šidák's multiple comparisons test.

	UNIVARIABLE				MULTIVARIABLE			
**Variable**	**Beta coefficient**	**Lower 95% CI**	**Upper 95% CI**	**P-value**	**Beta coefficient**	**Lower 95% CI**	**Upper 95% CI**	**P-value**
Duration of symptoms	0.583	0.042	1.124	0.035	0.851	0.331	1.37	0.002
Indexed left ventricular mass	7.012	3.932	10.093	0	4.311	1.364	7.258	0.005
Collagen fractional volume	0.054	0.022	0.086	0.001	5.662	1.003	10.32	0.018
Left bundle branch block	123.604	55.325	191.882	0.001	83.6	13.1214	154.076	0.021
QTc duration	1.166	0.418	1.914	0.003	0.801	0.078	1.524	0.031
Left Ventricular Ejection Fraction	-6.476	-10.514	-2.438	0.002				
Indexed Left Ventricular End systolic volume	1.227	0.26	2.194	0.014				
Indexed Left Ventricular End diastolic volume	1.086	0.143	2.029	0.025				

Table S2. Univariable and multivariable linear regression analysis of *SORBS2* expression in human heart failure patients. Duration of symptoms were the number months that a patient experienced symptoms before the biopsy for RNA-sequencing was taken. CI: confidence interval. Based on n = 67-72 biological replicates.

	UNIVARIABLE				MULTIVARIABLE			
Variable	Beta coefficient	Lower 95% CI	Upper 95% CI	P-value	Beta coefficient	Lower 95% CI	Upper 95% CI	P-value
Collagen expression	4.782	3.584	5.98	<0,001	2.994	2.097	3.89	<0,001
Left ventricular posterior wall end diastole	0.219	0.125	0.313	<0,001	0.095	0.008	0.182	0.034
Interventricular septal end systole	0.173	0.088	0.258	<0,001	0.09	0.012	0.168	0.025
Left ventricular mass corrected	0.001	0.001	0.001	<0,001	0.001	0	0.002	0.019
HW/TL	0.02	0.017	0.024	<0,001	0.01	0.002	0.017	0.01
Left ventricular end systolic volume	0.002	0.002	0.003	<0,001	0.002	0.001	0.003	0.002
TAB	0.096	0.067	0.126	<0,001				
Interventricular septal end diastole	0.23	0.152	0.307	<0,001				
Left ventricular internal dimension end diastole	0.089	0.05	0.128	<0,001				
Left ventricular internal dimension end systole	0.079	0.053	0.106	<0,001				
Ejection Fraction	-0.004	-0.005	-0.003	<0,001				
Fractional shortening	-0.006	-0.009	-0.004	<0,001				
Left ventricular mass	0.001	0.001	0.001	<0,001				
Left ventricular end diastolic volume	0.002	0.001	0.003	<0,001				

Table S3. Univariable and multivariable linear regression analysis of *Sorbs2* expression in mouse TAB timeline. CI: confidence interval. TAB: transverse aortic banding. HW/TL: heart weight to tibia length ratio. Based on n = 53 biological replicates.

Protein ID's	Protein names	Gene names	FDR bait	FDR condition	FDR interaction
P28660	Nck-associated protein 1	Nckap1	2.66E-13	0.247315786	0.66971322
Q8BH43;Q8R5H6	Wiskott-Aldrich syndrome protein family member 2	Wasf2	7.20E-13	0.366056524	0.730624283
Q7TMB8	Cytoplasmic FMR1-interacting protein 1	Cyfip1	1.69E-12	0.136865703	0.775245301
Q3UQ28	Peroxidasin homolog	Pxdn	1.21E-11	0.022788168	0.569420309
Q8CBW3	Abl interactor 1	Abi1	1.55E-11	0.286848495	0.740104149
Q3UTJ2	Sorbin and SH3 domain-containing protein 2	Sorbs2	5.32E-11	0.442223861	0.596015352
Q62523	Zyxin	Zyx	3.82E-10	0.170569566	0.794426319
Q91VR8	Protein BRICK1	Brk1	2.20E-09	0.40551729	0.757364025
Q8BYZ1	ABI gene family member 3	Abi3	7.67E-09	0.354788341	0.75869328
Q6P6M7	O-phosphoseryl-tRNA(Sec) selenium transferase	Sepsecs	9.52E-09	0.009990688	0.569420309
P62484	Abl interactor 2	Abi2	6.13E-08	0.147210848	0.77242465
O55101	Synaptogyrin-2	Syngr2	1.29E-07	0.025806294	0.686624883
Q64727	Vinculin	Vcl	1.18E-06	0.14202284	0.792865253
O35609	Secretory carrier-associated membrane protein 3	Scamp3	7.72E-06	0.063242401	0.774032744
Q5SQX6	Cytoplasmic FMR1-interacting protein 2	Cyfip2	8.29E-05	0.009781202	0.569420309
Q9WU78	Programmed cell death 6-interacting protein	Pdcd6ip	0.000166613	0.028214347	0.775245301
P68510	14-3-3 protein eta	Ywhah	0.001315912	0.423298307	0.569420309
P63101	14-3-3 protein zeta/delta	Ywhaz	0.00224148	0.187963856	0.746020456
Q9QZI9	Serine incorporator 3	Serinc3	0.00354596	0.369686914	0.676686207
P35979	60S ribosomal protein L12	Rpl12	0.004248655	0.255108672	0.569420309
P97765	WW domain-binding protein 2	Wbp2	0.004248655	0.069215225	0.750169763
Q8K1X4		Nckap1l	0.004606992	0.400830526	0.569420309
P63024;Q62442	Vesicle-associated membrane protein 3	Vamp3	0.004700325	0.442392599	0.754605159
P61979	Heterogeneous nuclear ribonucleoprotein K	Hnrnpk	0.005276488	0.017038767	0.569420309
P62259	14-3-3 protein epsilon	Ywhae	0.006719506	0.370194402	0.569420309
O09117	Synaptophysin-like protein 1	Sypl1	0.00744315	0.101836457	0.648691463
Q68FE2	Autophagy-related protein 9A	Atg9a	0.010299843	0.413789749	0.792194683

Q60631	Growth factor receptor-bound protein 2	Grb2	0.011392249	0.063242401	0.569420309
Q9JLV1	BAG family molecular chaperone regulator 3	Bag3	0.011392249	0.058302937	0.722423225
Q9CQU3	Protein RER1	Rer1	0.011530264	0.224089437	0.620045164
P68254	14-3-3 protein theta	Ywhaq	0.011748143	0.424962324	0.699307131
Q6VN19	Ran-binding protein 10	Ranbp10	0.013444834	0.315776063	0.580576132
P43275;P15864;P43274;P43277	Histone H1.1;Histone H1.2;Histone H1.4;Histone H1.3	Hist1h1a;Hist1h1c;Hist1h1e;Hist1h1d	0.014295115	0.234162077	0.569420309
O55028	[3-methyl-2-oxobutanoate dehydrogenase [lipoamide]] kinase, mitochondrial	Bckdk	0.015338251	0.028125624	0.746342951
Q80WQ2	Protein VAC14 homolog	Vac14	0.017368803	0.085266795	0.569420309
Q8VBT1;Q8BHN1;Q6PAM1	Beta-taxilin	Txlnb	0.017368803	0.222087629	0.569420309
P59017	Bcl-2-like protein 13	Bcl2l13	0.018384351	0.200779197	0.730624283
Q8K021	Secretory carrier-associated membrane protein 1	Scamp1	0.019781307	0.042892895	0.569420309
Q8R191	Synaptogyrin-3	Syngr3	0.019781307	0.030658367	0.569420309
Q9CR62	Mitochondrial 2-oxoglutarate/malate carrier protein	Slc25a11	0.02212104	0.031344563	0.767719919
B2RQC6	CAD protein;Glutamine-dependent carbamoyl-phosphate synthase;Aspartate carbamoyltransferase;Dihydroorotase	Cad	0.026817845	0.023356168	0.569420309
Q9JK92	Heat shock protein beta-8	Hspb8	0.027232936	0.219972552	0.668460289
O55100	Synaptogyrin-1	Syngr1	0.030391158	0.444506288	0.726487985
P62082	40S ribosomal protein S7	Rps7	0.039162951	0.424046848	0.742109152
Q6P549;Q9ES52;Q3TW96	Phosphatidylinositol 3,4,5-trisphosphate 5-phosphatase 2	Inppl1	0.040141897	0.406054652	0.569420309
P53986	Monocarboxylate transporter 1	Slc16a1	0.041856545	0.015381523	0.795295339
Q5FW52	Muscular LMNA-interacting protein	Mlip	0.045231062	0.142585827	0.676686207
Q5ND29	Rab-interacting lysosomal protein	Rilp	0.045231062	0.252823968	0.745435831

Table S4. All significant SORBS2 binding proteins.

TIMEPOINT	GENOTYPE	CONDITION	BASELINE	2 WEEKS	8 WEEKS	11 WEEKS	16 WEEKS
IVS;d (mm)	*Sorbs2* WT/WT	sham	0.77 ± 0.10	0.88 ± 0.11	0.92 ± 0.17	0.96 ± 0.16	0.97 ± 0.16
		TAB	0.82 ± 0.13	1.08 ± 0.18	1.21 ± 0.25	1.14 ± 0.14	1.27 ± 0.23
	Sorbs2 LoxP/LoxP	sham	0.93 ± 0.16*	0.88 ± 0.15	0.97 ± 0.27	0.97 ± 0.18	0.98 ± 0.28
		TAB	0.83 ± 0.09	1.33 ± 0.21*	1.51 ± 0.14*	1.35 ± 0.27	1.33 ± 0.43
IVS;s (mm)	*Sorbs2* WT/WT	sham	1.17 ± 0.17	1.35 ± 0.10	1.39 ± 0.16	1.38 ± 0.22	1.43 ± 0.20
		TAB	1.18 ± 0.18	1.54 ± 0.18	1.66 ± 0.24	1.57 ± 0.19	1.73 ± 0.25
	Sorbs2 LoxP/LoxP	sham	1.23 ± 0.21	1.33 ± 0.09	1.41 ± 0.29	1.42 ± 0.20	1.37 ± 0.32
		TAB	1.17 ± 0.08	1.79 ± 0.27*	1.98 ± 0.21*	1.92 ± 0.26*	1.91 ± 0.36
LVID;d (mm)	*Sorbs2* WT/WT	sham	4.20 ± 0.36	4.29 ± 0.33	4.36 ± 0.23	4.15 ± 0.36	4.41 ± 0.32
		TAB	4.05 ± 0.29	3.87 ± 0.44	4.31 ± 0.28	4.45 ± 0.50	4.56 ± 0.55
	Sorbs2 LoxP/LoxP	sham	4.07 ± 0.27	4.08 ± 0.27	4.04 ± 0.58	4.26 ± 0.27	4.09 ± 0.85
		TAB	4.16 ± 0.25	3.40 ± 0.56*	3.34 ± 0.37*	3.36 ± 0.51*	2.83 ± 0.66*
LVID;s (mm)	*Sorbs2* WT/WT	sham	3.07 ± 0.35	3.02 ± 0.35	3.06 ± 0.19	2.80 ± 0.41	3.12 ± 0.24
		TAB	2.93 ± 0.37	2.84 ± 0.51	3.31 ± 0.33	3.50 ± 0.55	3.58 ± 0.69
	Sorbs2 LoxP/LoxP	sham	3.01 ± 0.21	2.91 ± 0.23	2.89 ± 0.32	3.02 ± 0.37	2.99 ± 0.35
		TAB	3.09 ± 0.25	2.29 ± 0.58*	2.18 ± 0.39*	2.01 ± 0.43*	1.39 ± 0.72*
LVPW;d (mm)	*Sorbs2* WT/WT	sham	0.75 ± 0.07	0.91 ± 0.49	0.90 ± 0.14	0.84 ± 0.10	0.90 ± 0.14
		TAB	0.78 ± 0.10	1.13 ± 0.29	1.20 ± 0.22	1.17 ± 0.21	1.16 ± 0.20
	Sorbs2 LoxP/LoxP	sham	0.80 ± 0.08	0.86 ± 0.12	0.90 ± 0.18	0.85 ± 0.08	0.89 ± 0.09
		TAB	0.76 ± 0.05	1.46 ± 0.47	1.73 ± 0.71*	1.75 ± 0.70*	2.47 ± 0.63*
LVPW;s (mm)	*Sorbs2* WT/WT	sham	1.11 ± 0.08	1.31 ± 0.65	1.27 ± 0.12	1.30 ± 0.20	1.27 ± 0.22
		TAB	1.12 ± 0.10	1.47 ± 0.30	1.45 ± 0.27	1.44 ± 0.21	1.44 ± 0.22
	Sorbs2 LoxP/LoxP	sham	1.10 ± 0.06	1.20 ± 0.14	1.27 ± 0.20	1.23 ± 0.15	1.29 ± 0.11
		TAB	1.10 ± 0.07	1.86 ± 0.51	2.16 ± 0.67*	2.22 ± 0.63	3.07 ± 0.52*
EF(%)	*Sorbs2* WT/WT	sham	52.48 ± 9.64	56.99 ± 6.88	57.16 ± 5.06	61.03 ± 8.51	56.11 ± 5.24
		TAB	53.96 ± 7.34	52.72 ± 10.32	46.54 ± 7.90	43.87 ± 7.11	44.35 ± 10.00
	Sorbs2 LoxP/LoxP	sham	49.74 ± 7.51	55.53 ± 6.31	59.23 ± 6.29	56.42 ± 7.63	59.09 ± 5.76
		TAB	50.89 ± 4.89	62.40 ± 12.54*	65.26 ± 7.98*	71.74 ± 7.98*	81.18 ± 13.79*
FS (%)	*Sorbs2* WT/WT	sham	26.93 ± 5.93	29.81 ± 4.59	29.89 ± 3.40	32.63 ± 5.83	29.22 ± 3.51
		TAB	27.70 ± 4.73	26.95 ± 6.55	23.22 ± 4.64	21.65 ± 3.98	22.10 ± 5.67
	Sorbs2 LoxP/LoxP	sham	25.02 ± 4.54	28.73 ± 4.53	31.27 ± 4.42	29.44 ± 4.91	31.21 ± 3.89
		TAB	25.70 ± 3.02	33.69 ± 9.74*	35.13 ± 5.75*	40.39 ± 6.68	51.94 ± 17.43*

LV mass (mg)	*SORBS2* WT/WT	SHAM	121.57 ± 30.54	146.07 ± 37.63	163.03 ± 31.46	147.61 ± 30.52	170.18 ± 22.74
		TAB	120.23 ± 25.01	177.58 ± 30.66	237.74 ± 55.50	234.98 ± 55.54	267.39 ± 72.00
	Sorbs2 LoxP/LoxP	sham	132.62 ± 24.66	136.40 ± 20.12	158.13 ± 34.16	157.01 ± 28.03	165.64 ± 33.93
		TAB	123.84 ± 13.04	208.74 ± 50.48	265.47 ± 88.41	251.98 ± 110.98	294.82 ± 135.11
LV mass corrected (mg)	*Sorbs2* WT/WT	sham	97.26 ± 24.43	116.86 ± 30.10	130.42 ± 25.17	118.09 ± 24.42	136.14 ± 18.19
		TAB	96.18 ± 20.01	142.07 ± 24.53	190.19 ± 44.40	187.98 ± 44.43	213.91 ± 57.60
	Sorbs2 LoxP/LoxP	sham	106.10 ± 19.73	109.12 ± 16.09	126.51 ± 27.33	125.61 ± 22.43	132.51 ± 27.14
		TAB	99.07 ± 10.43	166.99 ± 40.39	212.38 ± 70.72	201.59 ± 88.78	235.86 ± 108.09
LV vol;d (μl)	*Sorbs2* WT/WT	sham	79.32 ± 16.13	83.25 ± 15.34	86.19 ± 10.50	77.03 ± 16.34	88.79 ± 15.30
		TAB	72.51 ± 12.77	64.53 ± 17.41	83.95 ± 12.85	91.47 ± 24.23	97.92 ± 26.44
	Sorbs2 LoxP/LoxP	sham	70.82 ± 8.58	74.41 ± 12.38	78.82 ± 12.52	82.02 ± 13.10	85.92 ± 18.34
		TAB	77.03 ± 11.21	49.23 ± 16.68*	46.12 ± 11.24*	47.48 ± 16.84*	32.57 ± 19.26*
LV vol;s (μl)	*Sorbs2* WT/WT	sham	37.69 ± 10.48	36.16 ± 10.23	36.83 ± 5.58	30.57 ± 10.80	38.83 ± 6.83
		TAB	33.96 ± 10.33	31.39 ± 13.96	45.20 ± 10.86	52.70 ± 19.93	56.61 ± 25.18
	Sorbs2 LoxP/LoxP	sham	35.52 ± 6.16	32.85 ± 6.26	32.47 ± 8.33	36.31 ± 11.28	35.40 ± 10.30
		TAB	37.97 ± 7.29	19.75 ± 9.64*	16.61 ± 6.87*	13.84 ± 7.67*	7.30 ± 9.83*

Table S5. Functional measurements at different timepoint after transverse aortic banding (TAB). IVS: inter ventricular septal thickness at diastole (;d) or systole (;s), LVID: left ventricular internal dimension at diastole (;d) or systole (;s), LVPW, left ventricular posterior wall thickness at diastole (;d) or systole (;s), EF: ejection fraction, FS: fractional shortening, LV mass: left ventricular mass derived from echocardiography analysis, LV mass corrected: Corrected LV mass derived from echocardiography analysis, LV vol;d: left ventricular end diastolic volume, LV vol;s: left ventricular end systolic volume, HW/BW: heart weight to body weight ratio, HW/TL: heart weight to tibia length ratio. Two-way ANOVA with Šidák's multiple comparisons test comparison for comparison against corresponding wildtype at each timepoint, *p<005. ± indicates standard deviation.

Spatial transcriptomics	Biological input	GSM
Visium	Mouse tissue 3 days post myocardial infarction	GSM6613084
Visium	Mouse tissue 3 days post myocardial infarction	GSM6613085
Visium	Mouse tissue 3 days post myocardial infarction	GSM6613086
Visium	Mouse tissue 7 days post myocardial infarction	GSM6613087
Visium	Mouse tissue 7 days post myocardial infarction	GSM6613088
Visium	Mouse tissue 7 days post myocardial infarction	GSM6613089
Visium	Mouse tissue post sham and mouse tissue post needle pass	GSM6613080
Visium	Mouse tissue post sham and mouse tissue post needle pass	GSM6613081

Table S6. Reanalyzed publicly available spatial transcriptomic data.

ChIP-seq	Biological input	PMID	SRA	SRA input
H3K27ac	human iPS-derived cardiomyocytes	27984724	SRR4032227;SRR4032199;SRR4032191;SRR4032207	SRR4032231
GATA4	human iPS-derived cardiomyocytes	27984724	SRR4032227;SRR4032199;SRR4032191;SRR4032207	SRR4032231
H3K27ac	mouse primary adult heart tissue	25249388	SRR1025227;SRR1550904	SRR1025232
GATA4	mouse primary adult heart tissue	25249388	SRR1025220;SRR1025221	SRR1025224

Table S7. Reanalyzed publicily available ChIP-seq data. hiPSC: human induced pluripotent stem cell.

**Oligonucleotide**	**Species**	**Sequence (5’ to 3’)**
RPL32 fw	Human	CAACGTCAAGGAGCTGGAAG
RPL32 rv	Human	TGGGGTTGGTGACTCTGATG
SORBS2 TSS-2 fw	Human	GGAAGCAGGTAAACCCGAGA
SORBS2 TSS-2 rv	Human	GAGCGGCAGGTCTGAAAAAG
Gapdh fw	Mouse	TGTCGTGGAGTCTACTGGTG
Gapdh rv	Mouse	ACACCCATCACAAACATGG
Polr2a fw	Mouse	CGGCCTGAGTGGATGATTGT
Polr2a rv	Mouse	CTGGTTTCGAGCAGAACCCT
Sorbs2 all variants	Mouse	CTCCAGACAGAAAGGTGGACA
Sorbs2 all variants	Mouse	ACGACTTCCCAGGCTCATATT
Sorbs2 TSS1 fw - pair 1	Mouse	CGGATGAACAACGCATAGACG
Sorbs2 TSS1 rv - pair 1	Mouse	TCTTAGATACGGGGGTGCTG
Sorbs2 TSS1 fw - pair 2	Mouse	CGGATGAACAACGCATAGACG
Sorbs2 TSS1 rv - pair 2	Mouse	TCTTAGATACGGGGGTGCTG
Sorbs2 TSS2 fw	Mouse	CGCTCTAGTGTTCACCTGCT
Sorbs2 TSS2 rv	Mouse	GCAGCTTCAGCGTGTAGGA
Sorbs2 TSS3a fw	Mouse	TGCCTGTGGTCTTAAGAAGAGG
Sorbs2 TSS3a rv	Mouse	ACAACCGGTCTTAGTGCAACA
Sorbs2 TSS3b fw	Mouse	CGGGGAAGTCAGCTTCTCTC
Sorbs2 TSS3b rv	Mouse	CCTTTAACGACCAGGTCTCAT
Sorbs2 TSS5 fw	Mouse	ATGTGTCCCTCTGGGTCTGT
Sorbs2 TSS5 rv	Mouse	GCTCCGAGGACAATCTTTATGC
Col1a1 fw	Mouse	AATGCAATGAAGAACTGGACTG
Col1a1 rv	Mouse	CCCTCGACTCCTACATCTTCTG
Col1a2 fw	Mouse	CAAGGACCTGCTGGTGAAC
Col1a2 rv	Mouse	TGGTCCAACGACTCCTCTC
Col3a1 fw	Mouse	GATGGCAAAGATGGATCACCTGG
Col3a1 rv	Mouse	GACCCTTTTCTCCTGGGATGC
Nppa fw	Mouse	GGTAGGATTGACAGGATTGGAG
Nppa rv	Mouse	GCTTAGGATCTTTTGCGATCTG
Nppb fw	Mouse	GAGTCCTTCGGTCTCAAGGC
Nppb rv	Mouse	CAACTTCAGTGCGTTACAGC
Xirp2 fw	Mouse	GCTTCTCGGCTAATGTCATGGA
Xirp2 rv	Mouse	TGACTGCTGTGGATTGCCTC
Acta1 fw	Mouse	CTATTCCTTCGTGACCACAG
Acta1 rv	Mouse	CCCAGAACTCAACACGATG
Myh7 fw	Mouse	TGACGCAGGAGAGCATCAT
Myh7 rv	Mouse	GAGTGCATTTAACTCAAAGTCCTTC
Thbs1 fw	Mouse	GTTGCAAAGGGAGATGTCAATG
Thbs1 rv	Mouse	GTCAAGGGTAAGAAGGACGTTG
Vcan fw	Mouse	CATGACTTCCGCTGGACTGA
Vcan rv	Mouse	GCAAGTGTAGGTGAGGTGGT
Col8a1 fw	Mouse	GCCAGCCAAGCCTAAATGT
Col8a1 rv	Mouse	CAGAGTTCAGGGAAATGATGAA
Tnc fw	Mouse	ACGGCTACCACAGAAGCTG
Tnc rv	Mouse	ATGGCTGTTGTTGCTATGGCA
Itga9 fw	Mouse	CTGGAGCACTTCCACGACAA

Itga9 rv	Mouse	ACACAGCTCCAGGAGACTTTAC
ItgaV fw	Mouse	ACGTCCTCCAGGATGTTTCTCCT
ItgaV rv	Mouse	ACACAGCTCCAGGAGACTTTAC
P3h2 fw	Mouse	TTACGAGGCAATCGCAGATCA
P3h2 rv	Mouse	GGCGAGTTCCCTCACACATT
Nmrk2 fw	Mouse	AAACTCATCATAGGCATTGGAGG
Nmrk2 rv	Mouse	GTCCTGGGGCTTGAAGAAGT
Igfbp5 fw	Mouse	ACAAGAGAAAGCAGTGTAAGCC
Igfbp5 rv	Mouse	CCGTACTTGTCCACACACCA
Dok5 fw	Mouse	GCAGACGCCTAGGGATTTATCA
Dok5 rv	Mouse	CAGTCTCTTTGGACCCTTGCT
Muc1 fw	Mouse	TTTCGGCAGGTAATGGCAGT
Muc1 rv	Mouse	GTGGGGTGACTTGCTCCTAC
Col4a5 fw	Mouse	CCCAAGTGCACCAGCATAAC
Col4a5 rv	Mouse	AGGTCCCTTCAGACCGATTG

Table S8. Primer sequences used for RT-qPCR. Forward and reversed primers are indicated by fw and rv, respectively.

**shRNA oligonucleotide**	**Sequence (5’ to 3’)**
shRNA-nc fw	AA**GAAATGTACTGCGTGGAGA**TCAAGAC**TCTCCACGCAGTACATTTC**TTTTTTTG
shRNA-nc rv	CAAAAAAA**GAAATGTACTGCGTGGAGA**GTCTTGA**TCTCCACGCAGTACATTTC**TT
shRNA-SORBS2 - 1 fw	AA**CCCCGCATTACCCAGGGAT**TCAAGAC**ATCCCTGGGTAATGCGGGG**TTTTTTTG
shRNA-SORBS2 - 1 rv	CAAAAAAA**CCCCGCATTACCCAGGGAT**GTCTTGA**ATCCCTGGGTAATGCGGGG**TT
shRNA-SORBS2 - 2 fw	AA**CCAACAGACAAGGCATCTT**TCAAGAC**AAGATGCCTTGTCTGTTGG**TTTTTTTG
shRNA-SORBS2 - 2 rv	CAAAAAAA**CCAACAGACAAGGCATCTT**GTCTTGA**AAGATGCCTTGTCTGTTGG**TT

Table S9. shRNA sequences used. Target sequence in oligonucleotide is indicated in bold. Nc: negative control. Forward and reverse are indicated by fw and rv, respectively.

Supplementary methods

Below a more extensive and detailed description of the materials and methods used in this study. To provide the reader with all relevant information it also includes information from the main methods section.

Reanalysis of human cardiac single-cell sequencing data

Cell info and count data from GSE109816 and GSE121893 were downloaded from the Gene Expression Omnibus (GEO). Data was analyzed using R version 3.6.2 and Seurat v3.1[1]. Filtering in line with the original publication was applied[2]. As such, cells were excluded if they contained less than 500 genes per cell, more than 72% mitochondrial reads, more UMIs than 2 standard deviations from the mean of log10 from all UMIs. Next, mitochondrial genes were removed to prevent interference with downstream analysis. CMs from CM-enriched digestion were only included if cells contained more than 10.000 distinct UMIs. In order to remove technical variation and bias due to individuals rather than biological condition, data was analyzed using the Seurat SCTransform integration workflow[1,3]. Following data integration, we found that most of the variation was included in the first 7 principal components. Therefore, these were used for subsequent clustering using the following parameters: *min.dist* = 0.2 and *resolution* = 0.6. Log normalized values were used for differential expression and visualization of clusters. Based on enriched genes expressed in different clusters we could identify CM, EC, FB, Pericytes, SMC, monocytes and lymphocytes. We selected the CM clusters for further analysis. We selected CMs originating from the left ventricle for further expression and correlation analysis.

Reanalysis of mouse cardiac single-cell sequencing data

Cell info and count data from GSE120064 was downloaded from the GEO. Data was analyzed using R version 3.6.2 and Seurat v3.1[1]. After a Seurat Object was created, sctransform normalization was applied[3]. Next, the first 20 principal components and a resolution of 0.7 were used for clustering. Lognormalized values were used for differential expression and visualization of clusters. Based on enriched genes expressed in different clusters we could identify CM, EC, FB, LE, SMC, monocytes, B-cells, T-cells and EPC. We selected the CM clusters for further analysis. Next, we selected CMs originating from the apical region. We noticed several CMs to be highly increased in erythrocyte markers. Since erythrocytes do not contain a nucleus, these could contaminate other cells in the single-cell selection of the ICell8 system which is based on visualization of nuclei. To exclude any potential interference of erythrocyte contaminated cells we excluded cells enriched in erythrocyte markers. Following the subsetting of CM's, the SCTransform wrapper was again applied to the RNA assay and the first 7 PC's and a resolution of 0.4 were used for subclustering and visualization. DEG and *Nppa* correlated genes were calculated based on lognormalized values.

Reanalysis of spatial transcriptomics

Spatial transcriptomic samples were downloaded from GSE214611 (Table S6). Data was analyzed using R version 4.1.3 and Seurat v4.0.1 and closely resembled original publication[4]. Different Visium slides were integrated using the Seurat integration workflow based on canonical correlation analysis[1]. GSM6613086 was used as reference slice and *dims = 1:20* was applied. Also,

for using the FindNeighbors function, *dims = 1:20* was used. Using a resolution of 0.5 with the FindClusters function, we identified 12 cluters. The FindAllMarkers using the Spatial data slot was applied to identify enriched genes for each cluster. These genes were used to classify clusters into remote, border and infarct zone. Next, the FindAllMarkers function was used again to identify enriched genes for all 3 zones.

Reanalysis ChIP-sequencing data, visualization of CAGE transcription start site (CTSS) tracks and visualization of single-cell chromatin accessibility at *SORBS2* TSS-2

Publicly available ChIP-seq data (Table S7) were mapped to the hg38 and mm10 reference genome and processed using the 4DN ChIPseq pipeline (https://github.com/4dn-dcic/chip-seq-pipeline2, archived at https://zenodo.org/doi/10.5281/zenodo.10638603). P-val signal bigwigs were used for the generation of the coverage plots in R version 4.1.3 using the GenomicRanges and Rtracklayer R/Bioconductor packages version 1.44.0 and 1.52.0, respectively[5,6]. CTSS tracks integrated into the ZENBU genome browser as described elsewhere[7], were downloaded using a fixed bin size of 10 and expression binning set at mean. The generated BED files were subsequently used for coverage plots similar to the ChIP-seq data described above. For visualization of single-cell chromatin accessibility around *SORBS2* TSS2, the online source of the cis-element ATLAS (http://catlas.org/humanenhancer) [8] was employed at 11-12-2023.

Experimental animals

All animal studies were performed in accordance with institutional guidelines and with approval of the Animal Welfare Committee of the Royal Netherlands Academy of Arts and Sciences. All animal experiments conform to the guidelines from Directive 2010/63/EU of the European Parliament on the protection of animals used for scientific purposes. All mice used in the presented study were males of the C57Bl/6J background. Mice containing LoxP sites surrounding exon 12 of the Sorbs2 gene (Sorbs2$^{LoxP)}$ resulting in a null allele upon removal were purchased from The Jackson Laboratory (#028600). To generate cardiomyocyte-specific inducible *Sorbs2* knock-out mice, mice transgenic for MerCreMer under the alpha-myosin heavy chain promotor (αMHC-MerCreMer) were crossed with Sorbs2^{LoxP} mice. Animals were randomized for experimental condition and blinded where possible (the severe phenotype of Sorbs2 deletion under TAB induced stress prevented blinding possibilities)

<u>Tamoxifen treatment</u>

To induce Cre-dependent recombination resulting in (heterozygous) genetic deletion of *Sorbs2*, mice were intraperitoneally injected daily for 3 consecutive days with 30μg/g tamoxifen (Chaymen Chemical, #13258) dissolved in a final solution of corn oil and 10% ethanol. The first tamoxifen injection was directly post-surgery. Regardless of experimental group, all mice were treated with tamoxifen to rule out any bias from tamoxifen induced Cre activation.

<u>Transverse Aortic Banding (TAB) surgery and transthoracic echocardiography</u>

TAB was used to induce pressure overload in mice. Mice were injected subcutaneously with the analgesic Buprenorphine (0.05-0.1 mg/kg) at least 30 minutes before surgery. A second and third dose of Buprenorphine (0.05-0.1 mg/kg) were given approximately 8-12 hours and 24 hours post-surgery, respectively. Anesthesia was induced by a mix of Fentanyl (0.05mg/kg) -Midzolam

(5mg/kg) -Dex-medetomidine (0.125mg/kg). If required, 1-2% isoflurane was added as maintenance anesthesia. During surgery, mice were kept on a heating pad of 38-39°C. Mice were connected to a ventilator by placing a tube into the trachea and hair of the thorax and neck was removed using hair removal cream. Next, the skin was disinfected with Iodine and 70% ethanol. An incision was made left of the midline to gain access to the first intercostal space. Accession to the heart was created by retraction of the pectoral muscles, a cut of the intercostal muscle caudal to the first rib and the use of wound hooks to separate the thymus. Next, the first and second aorta branch were identified and a 6.0 silk suture was placed at this location surrounding the aorta and closed with a 26G needle between the suture and the aorta, resulting in a standardized constriction. After removal of the needle and replacement of the thymus, the rib cage was closed using a 5.0 silk suture followed by replacement of the pectoral muscles and skin closing using a wound clip. For sham surgery the same procedure with exception of the placement of the needle was performed. Echocardiography was performed using a Visual Sonic Ultrasound system with a 30mHz transducer (VisualSonics Inc., Toronto, Canada) while sedation of mice was induced using 3% isoflurane and maintained using 1.5% isoflurane. Echocardiographic data analysis was performed using Vevo Lab software (version 5.6.0) based on short axis view M-mode measurements. After the last echocardiographic measurement, hearts were collected. For RNA or protein analysis, hearts were after collection washed in PBS, snap-frozen in liquid nitrogen and stored at -80°C until further use. For histology, hearts were washed in PBS, fixated in 4% formaldehyde at room temperature for 48 hours and embedded in parrafin.

Human patient correlations

Patients with early-stage DCM were included from the Maastricht Cardiomyopathy Registry[9]. All individuals fulfilling the diagnosis of DCM, who underwent endomyocardial biopsies (EMB), and had RNA-sequencing data available from their EMB were included in the current study (n=95). Details regarding RNA isolation, sequencing and analysis are described in detail previously[10]. In short, total RNA was isolated from the EMB using a mirVana (Ambion) kit. The mRNA sequencing library was generated using TruSeq mRNA sample preparation kit (Illumina). Pooled libraries consisting of equal molar samples were sequenced on a high-output 75bp single read on the NextSeq500 (Illumina). Reads were aligned to the human hg38 reference genome using TopHat and exonic reads were summed per transcript, and normalized as counts per million reads (cpm). All patients underwent a complete diagnostic work-up, including genetic testing, echocardiography, cardiac MRI, EMB, and holter analysis.

Uni- and multivariable regression analysis

For our human cohort, univariable linear regression analysis was performed to test the association between every individual clinical parameter (n=42) and the *SORBS2* expression from the RNA sequencing data from the cardiac biopsy. Afterwards, all univariable clinical parameters that were significantly associated with *SORBS2* expression (p-value <0.05) were included in a multivariable linear regression analysis, in which only clinical parameters were retained that had a p-value of <0.05. The same approach was taken to test association between clinical parameters and relative *Sorbs2* expression from quantitative real-time PCR derived from our TAB timeline, in which a total of 17 inidividual clinical parameters were taken along in the univariable linear regression analysis. All statistical analyses were performed in R environment, version 4.0.4.

Western blot

Protein concentration of cardiac tissue was assessed using Bradford assay (Bio-Rad). Equal amounts of protein were loaded for SDS-PAGE and analyzed by Western Blotting. Antibodies used were against SORBS2 (Sigma-Aldrich, #SAB4200183 d1:350, or Proteintech, #24643-1-AP d1:350), GAPDH (Sigma-Aldrich, #MAB374 d1:5000). An additional sample was taken along on every gel to normalize against for quantification purposes. For Western Blot analysis of immunoprecipitation experiments we used antibodies against WAVE2 (Cell Signaling, #3659 d1:1000) and SORBS2 (Sigma-Aldrich, #SAB4200183 d1:350).

Quantitative real-time PCR

TRIzol reagent (Thermo Fisher, #15596018) was used to isolate total RNA from cells or (engineered) heart tissue according to manufacturer's instructions. Generation of cDNA by reverse transcription was done using the iScript cDNA Synthesis Kit (Bio-Rad, #1708891). SYBRgreen methodology (Bio-Rad) was applied with iQ SYBR Green Supermix (Bio-Rad, #1708880) to measure mRNA abundance. Primer sequences used for RT-qPCR are given in Table S8. *Gapdh, RPL32* and *GUS* were used as housekeeping genes. Collagen expression was calculated as a combined score of *Col1a1*, *Col1a2* and *Col3a1* expression (each variant determining 1/3th of the score) which comprise approximately 90% of total the cardiac collagens[11].

Immunohistochemistry and immunofluorescence

Immunohistochemistry was performed on paraffin-embedded cardiac tissue slices. Tissue slices were deparaffinized and rehydrated using incubations in xylene and an ethanol to water gradient. Antigen retrieval was performed by boiling in Tris-EDTA buffer (pH = 9.0) for 20 minutes. After cooling down, blocking was applied using 0.5% BSA in PBS for 30 minutes at room temperature and followed by primary antibody incubation overnight at 4°C. This was followed by washing in PBS with 0.4% Tween-20 and secondary antibody incubation for 1 hour at room temperature. Next, washing was performed again followed by mounting. Primary antibodies used in this study are rabbit anti-SORBS2 (Proteintech, #24643-1-AP d1:100), mouse anti-N-cadherin (Santa Cruz, #sc-8424 d1:50). Corresponding secondary antibodies used were Alexa Fluor™ 568 donkey anti-rabbit IgG (Thermo Fisher Scientific, #A10042 d1:500), Alexa Fluor™ 488 donkey anti-mouse IgG (Thermo Fisher #A28175 d1:500). A Leica TCS SPE confocal microscope was used to take images. Images were processed using Fiji software. Human SORBS2 immunohistochemistry was performed using the Benchmark ULTRA (Ventana Medical Systems). Antigen retrieval was applied by 32 minutes at 100°C using Cell Conditioner #1 (pH = 9.0) (Ventana Medical Systems, #950-224). Primary antibody (Sigma-Aldrich, #SAB4200183 d1:100) was incubated for 1 hour followed by visualization using Optiview diaminobenzidine IHC detection kit (Ventana Medical Systems, #760-700).

Hematoxylin and Eosin, Picro Sirius Red and Alcian blue stainings were performed on whole heart slices which were imaged using a slide scanner (Olympus, VS200). Whole ventricular fibrosis (based on positivity for Picro Sirius Red) and positivity for Alcian blue was determined using QuPath[12]. Alcian blue stains were performed using a 1% (w/v) Alcian blue solution for 10 minutes at pH 2.5 followed by rinsing and a counterstain with Neutral red. FITC-conjugated Lectin

from wheat (Sigma Aldrich, #L4895) was employed to detect ECM and used for the analysis of cardiomyocyte cross-sectional area. Cross sectional area was determined semi-automatically using Cellpose 2.0 and the LabelstoRois plugin in ImageJ[13-15]. A total of 543 to 1454 cells were measured per sample. Picro Sirius Red stainings were used for polarized light microscopy and imaged using a slide scanner (Olympus, VS200). For quantification of polarized light images, representative regions of the left ventricular free wall were manually selected using QuPath and exported to ImageJ. Background was subtracted using the Rolling Ball Background Subtraction function. The hue (H), saturation (S), and brightness (B) ranges were set using the Color Threshold function. The ranges were based on previous work[16]: red (H1-13, S10-255, B20-255), orange (H 14-25, S 10-255, B 20-255), yellow (H 26-52, S 10-255, B 20-255), and green (H 53-110, S 10-255, B 20-255). The overall birefringence (total of all colors) was set to 100% in order te establish the relative expression of the individual hues. In situ cell death detection kit (Roche, #12156792910) was used for TUNEL labelling according to the manufacturer's instructions. Whole heart slices which were imaged using a slide scanner (Olympus, VS200). Ventricular cell death (based on positivity for TUNEL and DAPI) was determined using QuPath.

Affinity purification mass spectrometry

Immunoprecipitation

Left ventricular tissue was cut into small pieces on ice and placed in a mild lysis buffer of 0.1% Tween-20 in PBS with a Protease Inhibitor cocktail (Roche, #11836170001). Tissue lysis was continued by placing the sample in lysis buffer on a rotating wheel at 4°C for 45 minutes. This was followed by centrifugation at 21000RCF at 4°C for 12 minutes after which the supernatant was collected and protein concentration measured using Bradford assay (Bio-Rad). Magnetic Dynabeads Protein G (Thermo Fisher, #10003D) were coupled to a SORBS2 (Sigma-Aldrich, #SAB4200183) or WAVE2 (Cell Signaling, #3659) antibody or isotype control antibody (Thermo Fisher, #14-4714-85 or #02-6102) by rotation for 20 minutes at room temperature. After the magnetic beads were coupled to the antibody, beads were washed in a washing buffer of 0.02% Tween-20 in PBS with Protease Inhibitor cocktail. 1.25mg (SORBS2 IP) or 1.75mg (WAVE2 IP) of protein of each sample was added to 50μl of antibody coupled beads (SORBS2, d1:600 based on weight; WAVE2, d1:1167 based on weight) and incubated overnight at 4°C. The next day, beads were washed twice with washing buffer, resuspended in 100μl of washing buffer and transferred to a clean tube. Supernatant was removed again and beads were resuspended in 40μL of 4x XT Sample buffer (Bio-Rad, #1610791) diluted 1x in PBS. Next, immunoprecipitated proteins were eluted from the magnetic beads by incubation for 10 minutes at 70°C.

SDS PAGE and digestion of co-IP samples

Samples were loaded on a gel and ran for 2-3 cm and subsequently stained with colloidal coomassie dye G-250 (Thermo Fisher, #24590). Gel pieces were reduced, alkylated and digested overnight with trypsin at 37°C. Peptides were extracted with 100% acetonitrile (ACN) and dried in a vacuum concentrator. Samples were resuspended in 10% (v/v) formic acid for UHPLC-MS/MS analysis.

Mass spectrometry: RP-nanoLC-MS/MS

Resuspended peptides were subjected to LC-LC MS/MS using an Thermo Ultimate 3000 coupled to an Orbitrap Exploris 480 mass spectrometer (Thermo Scientific, Bremen, Germany) using a 46 minute gradient. Peptides were first trapped on a C18 PepMAP, 5 μm, 5 mm × 300 μm (Thermo Fisher, #160454) using solvent A (0.1% formic acid) before being separated on an analytical column (Agilent Poroshell EC-C18, 2.7 μm, 50 cm × 75 μm). The gradient was as follows: 9−13% solvent B (0.1 formic acid in 80% ACN) in 1 minute, 13−44% in 37 minute, 44-99% in 3 minute, 99% for 4 minute and back to 9% in 1 minute. The mass spectrometer was operated in data-dependent mode. Full-scan MS spectra from m/z 375−1600 were acquired at a resolution of 60 000 at m/z 200 after accumulation to the standard target value. Cycle time of 1 second was used with standard AGC targets. HCD fragmentation was performed at normalized collision energy of 28.

Analysis of mass spectrometry data

Protein identification was performed using MaxQuant 1.6.17.0.[17] by searching against the mus musculus proteome (https://www.uniprot.org, UP000000589, downloaded 19-07-2021) as reference database. A fasta file containing all reviewed canonical proteins was downloaded from https://www.uniprot.org. The database search was performed using default settings. The Andromeda search engine integrated into the MaxQuant environment was used to identify peptides by a target-decoy approach including a reversed database. For MaxQuant searches, default settings were applied: methionine oxidation and protein N-term acetylation as variable modifications. Trypsin with a maximum of two missed cleavages and a minimum peptide length of seven amino acids was set for enzyme specificity. A false discovery rate of 1% was used at protein, peptide and modification level. Unique and razor peptides were used. Maxquant output files were analyzed using Perseus 2.0.7.0[18]. After filtering for proteins classified as potential contaminants, only identified by site or reverse, iBAQ values were log2 transformed. Only proteins identified in at least 4 out of 6 SORBS2 pull-down samples in at least one group were included for further analysis. Missing values were imputed using the settings *Width = 0.4* and *Down shift = 1.8* and *Mode = Separately for each column*. Next, potential batch effects were corrected and values were normalized using a median subtraction and the *removeBatchEffect* function of the Limma package[19]. For statistical analysis a Two-Way ANOVA was performed for the SORBS2 IP which contained sham and TAB samples. After identification of significant interaction partners with an FDR <0.05, we removed IgG chain contaminants. The 48 significant interaction partners were uploaded to the STRING database for gene ontology and network analysis[20]. The network type was set at physical subnetwork.

RNA-sequencing

Library preparation and sequencing

RNA quality of starting material was checked with the Agilent Fragment Analyzer 5300 system using the RNA Kit (15nt) (Agilent, #DNF-471-1000). RNA quantity was determined using the Qubit RNA HS Assay Kit (Thermo Fisher, #Q32855) and an Invitrogen™ Qubit™ Fluorometer. TruSeq Stranded mRNA libraries (Illumina, #20020594) were prepared using a total of 100ng RNA according to the manufacturers protocol custom 384 xGen UDI-UMI adapters from IDT. Subsequently, libraries were checked using the Fragment Analyzer system dsDNA 910 Reagent Kit

(35-1500bp) (Agilent, #DNF-910-K1000) and the Qubit dsDNA HS Assay Kit (Thermo Fisher, #Q32854). Pooled equimolar sample libraries were then sequenced on an Illumina Nextseq2000 by using a P2 flowcell with 50bp paired-end reads.

Pre-processing of RNA-sequencing data

Quality control of sequencing reads from FASTQ files was performed using FastQC (v0.11.8). Reads were trimmed based on quality and adapter presence by TrimGalore (v0.6.5) followed by another quality check using FastQC. SortMeRNA (v4.3.3) was used to remove rRNA reads and remaining reads were aligned to using the STAR (v2.7.3a) aligner to the mouse reference genome (Mm_GRCm38_gatk_sorted.fasta). Mapped (bam) files were subjected to quality control using Sambamba (v0.7.0), RSeQC (v3.0.1) and PreSeq (v2.0.3). with the Mus_musculus.GRCm38.70.gtf gtf file serving as annotation to generate readcounts using the Subread FeatureCounts module (v2.0.0). We obtained 27.7 million assigned reads on average per sample.

Analysis of RNA-sequencing data

Raw counts were analysed using R version 4.1.3 and DESeq2 version 1.32.0[21]. We used the GSEA-MSigDB application to imply biological processes based on geometric normalized count tables derived from DESeq2[22]. Permutations was set at *geneset* and amount at 1000 (default). The default ranking method was applied, which is *signal to noise*. Genesets from the Mouse Molecular Signatures Database (MSigDB; 9-11-2022) were used: MH, M2: BioCarta subset of CP, Reactome subset of CP, WikiPathways subset of CP. M3: GTRD gene sets, M5: BP subset of GO, CC subset of GO, MF Subset of GO.

hiPS-CM culture

Human iPS-cells were obtained from ATCC (#ACS-1026). Differentiation into CMs was performed as described previously[23]. Briefly, iPS-cells were cultured up to a confluency of approximately 80-90% in Essential 8 Medium (Thermo Fisher, #A1517001) on Geltrex -coated plates (Thermo Fisher, #A1413302). Differentiation was started by replacing the medium with differentiation medium (RPMI medium (Thermo Fisher, #72400021) containing 0.5mg/ml recombinant human albumin (Sigma-Aldrich, #A9731), 0.2mg/ml L-ascorbic acid (Sigma-Aldrich, #A8960)). For the first two days, 4μM CHIR99021 (Sigma-Aldrich, #SML1046) was added to the differentiation medium. At day 2, the differentiation medium was refreshed and 5μM IWP2 (Millipore, #681671) was added until the medium was refreshed with differentiation medium at day 4 and day 6. At day 8, differentiation medium was removed and cardio-culture medium was added (RPMI medium (Thermo Fisher, #72400021) containing B-27 supplement (Thermo Fisher, #17504001)). Cardio-culture medium was refreshed every 2 days. At day 10 cardio-culture medium was replaced with selection medium to enhance purity (RPMI 1640 without glucose without HEPES (Biological Industries, #01-101-1A) containing 0.5mg/ml recombinant human albumin (Sigma-Aldrich, #A9731), 0.2mg/ml L-ascorbic acid (Sigma-Aldrich, A8960), 4mM lactate (Chemcruz, #SC-301818A) and 3.5mM HEPES (Sigma-Aldrich, #H0887). After selection was performed, cells were placed in cardio-culture medium with 1% penicillin-streptomcin (P/S) (Thermo Fisher, #15140122). For hiPS-CM experiment 16-20 day old cardiomyocytes (counted from day 0) were dissociated using 10 X TrypLE™ Select Enzyme (Gibco™, #A1217701) and plated in cardio-culture medium

with PS and 2μM Thiazovivin (Millipore, #420220) on geltrex-coated wells. 24 hours post plating, medium was refreshed to cardio-culture medium with PS.

siRNA and ET-1 treatment

hIPS-CMs were treated with 10nM of control siRNA (Thermo Fisher, #439084) or a siRNA targeting *Gata4* (Thermo Fisher, #4392420, ID: s535120) for 72 hours. siRNA transductions were performed using Opti-MEM (Gibco™, #11058021) and Lipofectamine™ RNAiMAX (Thermo Fisher, #13778075). ET-1 (Sigma-Aldrich, #E7764) or vehicle control (DMSO) treatment occurred at a concentration of 10nM 48 hours and again at 24 hours before collection.

hiPS-CM purity

Purity of hiPS-CMs was assessed by fluorescent activated cell sorting (FACS) using a BD LSRFortessa X-20. Cells were washed with PBS, dissociated using TripleSelect (Gibco, #A1217701) and collected by centrifugation for 5 minutes at 300G. After collection, cells were washed with PBS and fixated using ice-cold 70% ethanol and stored at 4°C until further use. Next, cells were permeabilized and blocked using blocking buffer (PBS, 5% FBS, 1%BSA, 0.5% Triton X-100). Cells were incubated with an antibody against Cardiac Troponin T (Abcam, #45932, d1:1000) in blocking buffer for 1 hour at room temperature. Cells were washed and incubated with a secondary antibody (Thermo Fisher, Alexa Fluor 568, #A10042, d1:500) and DAPI (Invitrogen, #D3571, d1:1000) in blocking buffer for 30 minutes at room temperature. Cells were collected in a tube with a strainer cap (Falcon, #352235) and purity was assessed by Cardiac Troponin T positive cells (cells stained with only the secondary antibody were used as negative control) analyzed using a BD LSRFortessa X-20 FACS machine.

Immunoprecipitation hiPS-CMs

hiPS-CM cells were washed with PBS and collected using Versene (Gibco™, #15040066). Cells were centrifuged for 3 minutes at 750G after which the supernatant was removed. Then, cells were resuspended in a mild lysis buffer of 0.1% Tween-20 in PBS with a Protease Inhibitor cocktail (Roche, #11836170001) which is the same lysis buffer for tissue immunoprecipitation experiments. From this point onwards, the immunoprecipitation steps are the same as described for the tissue immunoprecipitation experiments. The SORBS2 antibody (Sigma-Aldrich, #SAB4200183) dilution used was 1:80 (volume based). Equal quantity of isotype control antibody (Thermo Fisher, #14-4714-85) was used. 1% input was loaded for western blot analysis.

EHM generation

EHM's were produced using methodology described in a previous study[24]. In brief, a mixture of hiPS-CMs and human foreskin fibroblasts (HFF) (ATCC, HFF-1, #SCRC-1041) in a 7:3 ratio was resuspended in a Collagen type I solution (Collagen Solutions, #FS22024) that had been diluted in RPMI 2x (Thermo Fisher, #51800-035). This mixture was subsequently poured into wells of an EHM multi-well plate (Myriamed GmbH, myrPlate-TM5). After a 45 minute incubation at 37°C, EHM medium supplemented with TGFβ1 (Peprotech, #AF-100-21C) was introduced and changed daily for the initial 3-day period. Following this, medium was replaced daily with EHM medium for the entire duration of the experiment according to original protocol.

EHM contraction analysis

Contraction assessments were carried out by capturing video-optic recordings of EHM-mediated pole bending, utilizing the myrPlate-TM5 setup, at 37°C[25]. Spontaneously contracting EHM samples, were recorded for a minimum of 45 seconds at a frame rate of 50 fps, at specified time intervals, using a myrImager prototype developed by myriamed GmbH. The percentage of pole bending was quantified as an indicator of the force of contraction (F).

AAV-shRNA generation and transduction of hIPS-CMs

shRNAs targeting *SORBS2* were designed to target all isoforms of *SORBS2* (Table S9) and cloned into pAAV-U6-sgRNA-CMV-GFP[26] (Addgene, #85451) at the location of the gRNA scaffold such that the shRNA is transcribed from the U6 promotor. A non-targeting shRNA (Table S9) was used as negative control. Recombinant AAV9 vectors used in this study were prepared by the AAV Vector Unit at the International Centre for Genetic Engineering and Biotechnology Trieste (ICGEB) (http://www.icgeb.org/avu-core-facility.html), as described previously[27] with a few modifications. Briefly, infectious AAV vector particles were generated in HEK293T cells cultured in roller bottles by a three-plasmids transfection cross-packaging approach whereby the vector genome was packaged into AAV capsid serotype-9[28]. Purification of viral particles was obtained by PEG precipitation and two subsequent $CsCl_2$ gradient centrifugations[29]. The physical titer of recombinant AAVs was determined by absolute quantification of vector genomes (vg) packaged into viral particles, by real-time PCR using a standard curve of a plasmid containing the vector genome[30]; values obtained were above $1x10^{13}$ vg per milliliter. hiPS-CM were transduced with a mixture of 2 different AAV9-shRNAs targeting *SORBS2* or a non-targeting AAV9-shRNA at a total of 100k viral genomes per cell. The virus containing media was washed away before proceeding to EHM casting with HFF, which was performed 7 days post transduction.

Promotor activity assay

Sorbs2 promotor activity was determined using the Dual Luciferase Reporter® Assay System (Promega #E1910) and a Luminometer (Berthold Technologies, Centro XS[3] LB 960). A 451bp region (mm39 chr8:46080750-46081200) covering GATA4 ChIP-seq peak at *Sorbs2* TSS-2 was cloned into the pGL4.23[luc2/minP] vector (Promega, #E8411) using a Gibson Assembly® method. HEK293T cells were plated in a 24 well plate on day one. 24 hours later, cells were placed in serum free medium (DMEM Glutamax, Gibco™, #31966021) and transfected using a mixture of Opti-MEM (Gibco™, #11058021), Polyethylenimine (PEI) (Polysciences, #23966) and a total of 500ng DNA. For dose response assays, the transfected DNA mixture contained 0, 50, 150 or 300ng of a GATA4 expression plasmid, 150ng of pGL4.23[luc2/minP] containing the 451bp *Sorbs2* promotor region, 10ng of renilla control plasmid (pRL-CMV) (Promega, #E2261), and finally 340, 290, 190 or 40ng of the same backbone vector as the GATA4 expression plasmid, but containing mNeonGreen instead of GATA4. The latter was also used to verify successful transfection. Medium was refreshed 24 hours after transfection and cells were collected for read-out 48 hours after transfection using passive lysis according to manufacturer's guidelines. Using site directed mutagenesis the GATAA motif was mutated into GCCAA. To test the effect of this mutation, a similar approach as described above was used with the DNA mixture of 50ng of pGL4.23[luc2/minP] containing the 451bp *Sorbs2* promotor region with or without the mutated

site, 10ng of pRL-CMV, 50ng of the GATA4 expression plasmid, and finally 290ng of the control mNeonGreen expression plasmid.

Statistics

Statistical analysis of affinity purification mass spectrometry, RNA-seq and reanalysis of ChIP-seq data is described in detail above. All remaining statistical analyses were performed using Prism version 9.5.1. Student's and nested t-tests, as well as ANOVAs, were conducted after confirming that the necessary assumptions, such as normality and homoscedasticity, were met. In cases where these assumptions were not directly fulfilled, tests were applied to transformed data. If transformation did not suffice to meet the required assumptions, non-parametric tests were employed. In a few instances involving two-way ANOVA analyses, the prerequisites could not be met, and this is mentioned specifically in the corresponding legend. Each statistical test and/or multiple comparisons correction is mentioned in the corresponding figure and table legend.

References

1. Stuart T, Butler A, Hoffman P, Hafemeister C, Papalexi E, Mauck WM, 3rd, Hao Y, Stoeckius M, Smibert P, Satija R. Comprehensive Integration of Single-Cell Data. *Cell*. 2019;177:1888-1902.e1821. doi: 10.1016/j.cell.2019.05.031
2. Wang L, Yu P, Zhou B, Song J, Li Z, Zhang M, Guo G, Wang Y, Chen X, Han L, et al. Single-cell reconstruction of the adult human heart during heart failure and recovery reveals the cellular landscape underlying cardiac function. *Nat Cell Biol*. 2020;22:108-119. doi: 10.1038/s41556-019-0446-7
3. Hafemeister C, Satija R. Normalization and variance stabilization of single-cell RNA-seq data using regularized negative binomial regression. *Genome Biol*. 2019;20:296. doi: 10.1186/s13059-019-1874-1
4. Calcagno DM, Taghdiri N, Ninh VK, Mesfin JM, Toomu A, Sehgal R, Lee J, Liang Y, Duran JM, Adler E, et al. Single-cell and spatial transcriptomics of the infarcted heart define the dynamic onset of the border zone in response to mechanical destabilization. *Nat Cardiovasc Res*. 2022.
5. Lawrence M, Gentleman R, Carey V. rtracklayer: an R package for interfacing with genome browsers. *Bioinformatics*. 2009;25:1841-1842. doi: 10.1093/bioinformatics/btp328
6. Lawrence M, Huber W, Pagès H, Aboyoun P, Carlson M, Gentleman R, Morgan MT, Carey VJ. Software for computing and annotating genomic ranges. *PLoS Comput Biol*. 2013;9:e1003118. doi: 10.1371/journal.pcbi.1003118
7. Deviatiiarov RM, Gams A, Kulakovskiy IV, Buyan A, Meshcheryakov G, Syunyaev R, Singh R, Shah P, Tatarinova TV, Gusev O, et al. An atlas of transcribed human cardiac promoters and enhancers reveals an important role of regulatory elements in heart failure. *Nat Cardiovasc Res*. 2023. doi: https://doi.org/10.1038/s44161-022-00182-x
8. Zhang K, Hocker JD, Miller M, Hou X, Chiou J, Poirion OB, Qiu Y, Li YE, Gaulton KJ, Wang A, et al. A single-cell atlas of chromatin accessibility in the human genome. *Cell*. 2021;184:5985-6001.e5919. doi: 10.1016/j.cell.2021.10.024
9. Henkens M, Weerts J, Verdonschot JAJ, Raafs AG, Stroeks S, Sikking MA, Amin H, Mourmans SGJ, Geraeds CBG, Sanders-van Wijk S, et al. Improving diagnosis and risk

stratification across the ejection fraction spectrum: the Maastricht Cardiomyopathy registry. *ESC Heart Fail*. 2022;9:1463-1470. doi: 10.1002/ehf2.13833

10. Verdonschot JAJ, Merlo M, Dominguez F, Wang P, Henkens M, Adriaens ME, Hazebroek MR, Masè M, Escobar LE, Cobas-Paz R, et al. Phenotypic clustering of dilated cardiomyopathy patients highlights important pathophysiological differences. *Eur Heart J*. 2021;42:162-174. doi: 10.1093/eurheartj/ehaa841
11. McCabe MC, Saviola AJ, Hansen KC. Mass Spectrometry-Based Atlas of Extracellular Matrix Proteins across 25 Mouse Organs. *J Proteome Res*. 2023;22:790-801. doi: 10.1021/acs.jproteome.2c00526
12. Bankhead P, Loughrey MB, Fernández JA, Dombrowski Y, McArt DG, Dunne PD, McQuaid S, Gray RT, Murray LJ, Coleman HG, et al. QuPath: Open source software for digital pathology image analysis. *Sci Rep*. 2017;7:16878. doi: 10.1038/s41598-017-17204-5
13. Waisman A, Norris AM, Elías Costa M, Kopinke D. Automatic and unbiased segmentation and quantification of myofibers in skeletal muscle. *Sci Rep*. 2021;11:11793. doi: 10.1038/s41598-021-91191-6
14. Pachitariu M, Stringer C. Cellpose 2.0: how to train your own model. *Nat Methods*. 2022;19:1634-1641. doi: 10.1038/s41592-022-01663-4
15. Stringer C, Wang T, Michaelos M, Pachitariu M. Cellpose: a generalist algorithm for cellular segmentation. *Nat Methods*. 2021;18:100-106. doi: 10.1038/s41592-020-01018-x
16. Bauman TM, Nicholson TM, Abler LL, Eliceiri KW, Huang W, Vezina CM, Ricke WA. Characterization of fibrillar collagens and extracellular matrix of glandular benign prostatic hyperplasia nodules. *PLoS One*. 2014;9:e109102. doi: 10.1371/journal.pone.0109102
17. Cox J, Mann M. MaxQuant enables high peptide identification rates, individualized p.p.b.-range mass accuracies and proteome-wide protein quantification. *Nat Biotechnol*. 2008;26:1367-1372. doi: 10.1038/nbt.1511
18. Tyanova S, Temu T, Sinitcyn P, Carlson A, Hein MY, Geiger T, Mann M, Cox J. The Perseus computational platform for comprehensive analysis of (prote)omics data. *Nat Methods*. 2016;13:731-740. doi: 10.1038/nmeth.3901
19. Ritchie ME, Phipson B, Wu D, Hu Y, Law CW, Shi W, Smyth GK. limma powers differential expression analyses for RNA-sequencing and microarray studies. *Nucleic Acids Res*. 2015;43:e47. doi: 10.1093/nar/gkv007
20. Szklarczyk D, Franceschini A, Wyder S, Forslund K, Heller D, Huerta-Cepas J, Simonovic M, Roth A, Santos A, Tsafou KP, et al. STRING v10: protein-protein interaction networks, integrated over the tree of life. *Nucleic Acids Res*. 2015;43:D447-452. doi: 10.1093/nar/gku1003
21. Love MI, Huber W, Anders S. Moderated estimation of fold change and dispersion for RNA-seq data with DESeq2. *Genome Biol*. 2014;15:550. doi: 10.1186/s13059-014-0550-8
22. Subramanian A, Tamayo P, Mootha VK, Mukherjee S, Ebert BL, Gillette MA, Paulovich A, Pomeroy SL, Golub TR, Lander ES, et al. Gene set enrichment analysis: a knowledge-based approach for interpreting genome-wide expression profiles. *Proc Natl Acad Sci U S A*. 2005;102:15545-15550. doi: 10.1073/pnas.0506580102

23. Molenaar B, Timmer LT, Droog M, Perini I, Versteeg D, Kooijman L, Monshouwer-Kloots J, de Ruiter H, Gladka MM, van Rooij E. Single-cell transcriptomics following ischemic injury identifies a role for B2M in cardiac repair. *Commun Biol*. 2021;4:146. doi: 10.1038/s42003-020-01636-3
24. Tiburcy M, Hudson JE, Balfanz P, Schlick S, Meyer T, Chang Liao ML, Levent E, Raad F, Zeidler S, Wingender E, et al. Defined Engineered Human Myocardium With Advanced Maturation for Applications in Heart Failure Modeling and Repair. *Circulation*. 2017;135:1832-1847. doi: 10.1161/circulationaha.116.024145
25. Tiburcy M, Meyer T, Liaw NY, Zimmermann WH. Generation of Engineered Human Myocardium in a Multi-well Format. *STAR Protoc*. 2020;1:100032. doi: 10.1016/j.xpro.2020.100032
26. Duan Y, Ma G, Huang X, D'Amore PA, Zhang F, Lei H. The Clustered, Regularly Interspaced, Short Palindromic Repeats-associated Endonuclease 9 (CRISPR/Cas9)-created MDM2 T309G Mutation Enhances Vitreous-induced Expression of MDM2 and Proliferation and Survival of Cells. *J Biol Chem*. 2016;291:16339-16347. doi: 10.1074/jbc.M116.729467
27. Arsic N, Zacchigna S, Zentilin L, Ramirez-Correa G, Pattarini L, Salvi A, Sinagra G, Giacca M. Vascular endothelial growth factor stimulates skeletal muscle regeneration in vivo. *Mol Ther*. 2004;10:844-854. doi: 10.1016/j.ymthe.2004.08.007
28. Inagaki K, Fuess S, Storm TA, Gibson GA, McTiernan CF, Kay MA, Nakai H. Robust systemic transduction with AAV9 vectors in mice: efficient global cardiac gene transfer superior to that of AAV8. *Mol Ther*. 2006;14:45-53. doi: 10.1016/j.ymthe.2006.03.014
29. Ayuso E, Mingozzi F, Montane J, Leon X, Anguela XM, Haurigot V, Edmonson SA, Africa L, Zhou S, High KA, et al. High AAV vector purity results in serotype- and tissue-independent enhancement of transduction efficiency. *Gene Ther*. 2010;17:503-510. doi: 10.1038/gt.2009.157
30. Zentilin L, Marcello A, Giacca M. Involvement of cellular double-stranded DNA break binding proteins in processing of the recombinant adeno-associated virus genome. *J Virol*. 2001;75:12279-12287. doi: 10.1128/jvi.75.24.12279-12287.2001
